# Bidirectional Mendelian randomization study of psychiatric disorders and Parkinson’s disease

**DOI:** 10.3389/fnagi.2023.1120615

**Published:** 2023-03-14

**Authors:** Qi Wu, Shulin Liu, Xiurong Huang, Jiabin Liu, Yige Wang, Yaqing Xiang, Xuxiong Tang, Qian Xu, Xinxiang Yan, Beisha Tang, Jifeng Guo

**Affiliations:** ^1^Department of Neurology, Xiangya Hospital, Central South University, Changsha, Hunan, China; ^2^Key Laboratory of Hunan Province in Neurodegenerative Disorders, Central South University, Changsha, Hunan, China; ^3^Hunan International Scientific and Technological Cooperation Base of Neurodegenerative and Neurogenetic Diseases, Changsha, China; ^4^Center for Medical Genetics and Hunan Key Laboratory of Medical Genetics, School of Life Sciences, Central South University, Changsha, Hunan, China; ^5^Engineering Research Center of Hunan Province in Cognitive Impairment Disorders, Central South University, Changsha, China; ^6^National Clinical Research Center for Geriatric Disorders, Xiangya Hospital, Central South University, Changsha, Hunan, China

**Keywords:** Parkinson’s disease, psychiatric disorders, Mendelian randomization, genome-wide association studies, causal relationship

## Abstract

**Introduction:**

Although the relationship between psychiatric disorders and Parkinson’s disease (PD) has attracted continuous research attention, the causal linkage between them has not reached a definite conclusion.

**Methods:**

To identify the causal relationship between psychiatric disorders and PD, we used public summary-level data from the most recent and largest genome-wide association studies (GWASs) on psychiatric disorders and PD to conduct a bidirectional two-sample Mendelian randomization (MR). We applied stringent control steps in instrumental variable selection using the Mendelian randomization pleiotropy residual sum and outlier (MR-PRESSO) method to rule out pleiotropy. The inverse-variance weighted (IVW) method was used to identify the causal relationship between psychiatric disorders and PD. Multiple MR analysis methods, including MR-Egger, weighted-median, and leave-one-out analyses, were used for sensitivity analysis, followed by heterogeneity tests. Further validation and reverse MR analyses were conducted to strengthen the results of the forward MR analysis.

**Results:**

The lack of sufficient estimation results could suggest a causal relationship between psychiatric disorders and PD in the forward MR analysis. However, the subsequent reverse MR analysis detected a causal relationship between PD and bipolar disorder (IVW: odds ratios [OR] =1.053, 95% confidence interval [CI] =1.02–1.09, *p* = 0.001). Further analysis demonstrated a causal relationship between genetically predicted PD and the risk of bipolar disorder subtype. No pleiotropy or heterogeneity was detected in the analyses.

**Discussion:**

Our study suggested that while psychiatric disorders and traits might play various roles in the risk of developing PD, PD might also be involved in the risk of developing psychiatric disorders.

## Introduction

Parkinson’s disease (PD), the most common movement disorder and the second most common neurodegenerative disease, is characterised by a wide range of motor and non-motor symptoms (NMS) (Lees et al., 2009). PD affects 1% of the population over 60 years of age, and its prevalence generally ranges from 100 to 200 per 100,000 persons in unselected populations (von Campenhausen et al., 2005). Owing to PD aetiology, particularly in sporadic cases, distinguishing causal risk factors could be meaningful for treatment and prevention of this disease. PD diagnosis is mainly based on typical motor symptoms, including tremors, rigidity, bradykinesia, and motor impairment, which have also been increasingly considered an important component of PD (Armstrong and Okun, 2020). NMS, including sleep disorders, autonomic dysfunction, cognitive/neurobehavioural abnormalities, and sensory abnormalities (such as anosmia, pain, and paraesthesia) have an even higher negative impact on the quality of life than motor symptoms (Karlsen et al., 2000). In general, NMS are highly prevalent in PD patients; NMS can also develop at any phases of the disease and frequently precedes the onset of motor symptoms (Krishnan et al., 2011; Kim et al., 2013). Psychiatric disorders, including anxiety, depression, psychosis, sleep disturbances, and behavioural and cognitive changes, are widely recognised as common NMS in PD and are more frequent in PD patients than in the general population (Aarsland et al., 2009). Furthermore, numerous studies have shown that the morbidity of psychotic illnesses is higher in PD patients (Djamshidian and Friedman, 2014; Ghaddar et al., 2016; Ffytche et al., 2017).

Increasing evidence points to the multifactorial aetiology of PD, which involves ageing, genetic predisposition, environmental agents, trauma, and psychosocial impact (Perry et al., 2016). Additionally, psychiatric disorders have a significant influence on the quality of life of PD patients and may play a critical role in the rapid deterioration of clinical manifestations (Alvarado-Bolanos et al., 2015; Ng et al., 2015; Elefante et al., 2021). Therefore, further understanding of the association between psychiatric disorders and PD is valuable for research and clinical practice.

Psychiatric disorders have been proposed to precede PD development and be a possible PD risk factor, which has been proven in observational studies (Ishihara-Paul et al., 2008; Jacob et al., 2010; Sanyal et al., 2010; Lin et al., 2014; Gustafsson et al., 2015; Schrag et al., 2015). A meta-analysis of prospective studies using a large United Kingdom biobank indicated that neuroticism was consistently associated with a higher risk of PD (Terracciano et al., 2021). Another analysis of four cohort studies and three cross-sectional studies, consisting of 4,374,211 individuals, also reported that patients with bipolar disorder (BD) might be at risk of having a subsequent idiopathic PD diagnosis (Dols and Lemstra, 2020). Additionally, sleep disturbances, including insomnia and rapid eye movement sleep behaviour disorder (RBD), are often considered early markers of PD pathology and an independent PD risk factor (Hsiao et al., 2017). Although specific underlying mechanism of this phenomenon remains unclear, recent studies have indicated that this process could be related to abnormal brainstem α-synucleinopathy, which caused RBD (Bohnen and Hu, 2019). These studies suggested that psychiatric disorders might potentially trigger the onset of PD. With the global escalation of the ageing process, the rapidly increasing incidence of PD is impacting the quality of life of more people. Therefore, clarifying the causality of these associations could be of practical value for improving the health of the elderly.

Mendelian randomization (MR) is an epidemiological technique that investigates the causal relationship between risk factors and outcomes. MR tends to avoid confounding issues and reverse causality by using genetic variants as instrumental variables (IVs). This technique has been extensively used to validate causal relationships discovered in observational studies (Plotnikov and Guggenheim, 2019). Owing to the development of genomic techniques and methodologies, an increasing number of disease-associated variants have been detected by genome-wide association studies (GWAS), which provide sufficient IV resources that could be used to increase the power of MR. Therefore, we used publicly available GWAS summary-level data of psychiatric disorders and PD for a bidirectional two-sample MR analysis to validate and explore the relationships among these traits.

## Methods

### Data source

In this two-sample MR analysis, we included publicly available GWAS results of eight psychiatric disorders/traits and PD conducted in European populations. The basic characteristics of GWASs, including exposures and outcomes, are listed in Table 1. Exposure to interest included anorexia nervosa (AN) (Watson et al., 2019), anxiety disorder (Purves et al., 2020), BD (Mullins et al., 2021), insomnia (Watanabe et al., 2022), major depressive disorder (MDD) (Howard et al., 2019), neuroticism (Nagel et al., 2018), obsessive–compulsive disorder (OCD) [International Obsessive Compulsive Disorder Foundation Genetics Collaborative (LOCDF-GC) and OCD Collaborative Genetics Association Studies (OCGAS), 2018], and schizophrenia (Trubetskoy et al., 2022). For PD, we used the largest GWAS conducted in the European population (Nalls et al., 2019) as a discovery study and a GWAS conducted in the Finnish population (Kurki et al., 2023) as a validation study. Detailed information regarding genotype platforms, statistical analysis protocols, and participants for each study is available in the corresponding papers (Data presentation).

**Table 1:** Characteristics of GWASs used for each disorder.

Traits	Sample size (cases/controls)	Population	Consortium	PMID
**Exposure**				
Anorexia nervosa	72,517 (16,992/55,525)	Europeans	PGC*	31,308,545
Anxiety	83,565 (25,453/58,113)	Europeans	UKBB*	31,748,690
Bipolar disorder	413,466 (41,917/371,549)	Europeans	PGC	34,002,096
Insomnia	2,365,010 (593,724/1,771,286)	Europeans	CNCR*	30,804,565
MDD*	500,199 (170,756/329,443)	Europeans	PGC	30,718,901
Neuroticism	390,278^#^	Europeans	CNCR	29,942,085
OCD*	9,725 (2,688/7,037)	Europeans	PGC	28,761,083
Schizophrenia	130,644 (53,386/77,258)	Europeans	PGC	35,396,580
**Outcome**				
Parkinson’s disease	482,730 (33,674/449,056)	Europeans	IPDGC*	31,701,892
Parkinson’s disease	260,405 (2,496/257,909)	Finnish	FinnGen	www.finngen.fi

^*^MDD, major depressive disorder; OCD, obsessive–compulsive disorder; PGC, Psychiatric Genomics Consortium; UKB, UK Biobank; CNCR, Center for Neurogenomics and Cognitive Research; IPDGC, International Parkinson Disease Genomics Consortium. ^#^Weighted neuroticism sum-score.

### IV selection

**Figure 1:**
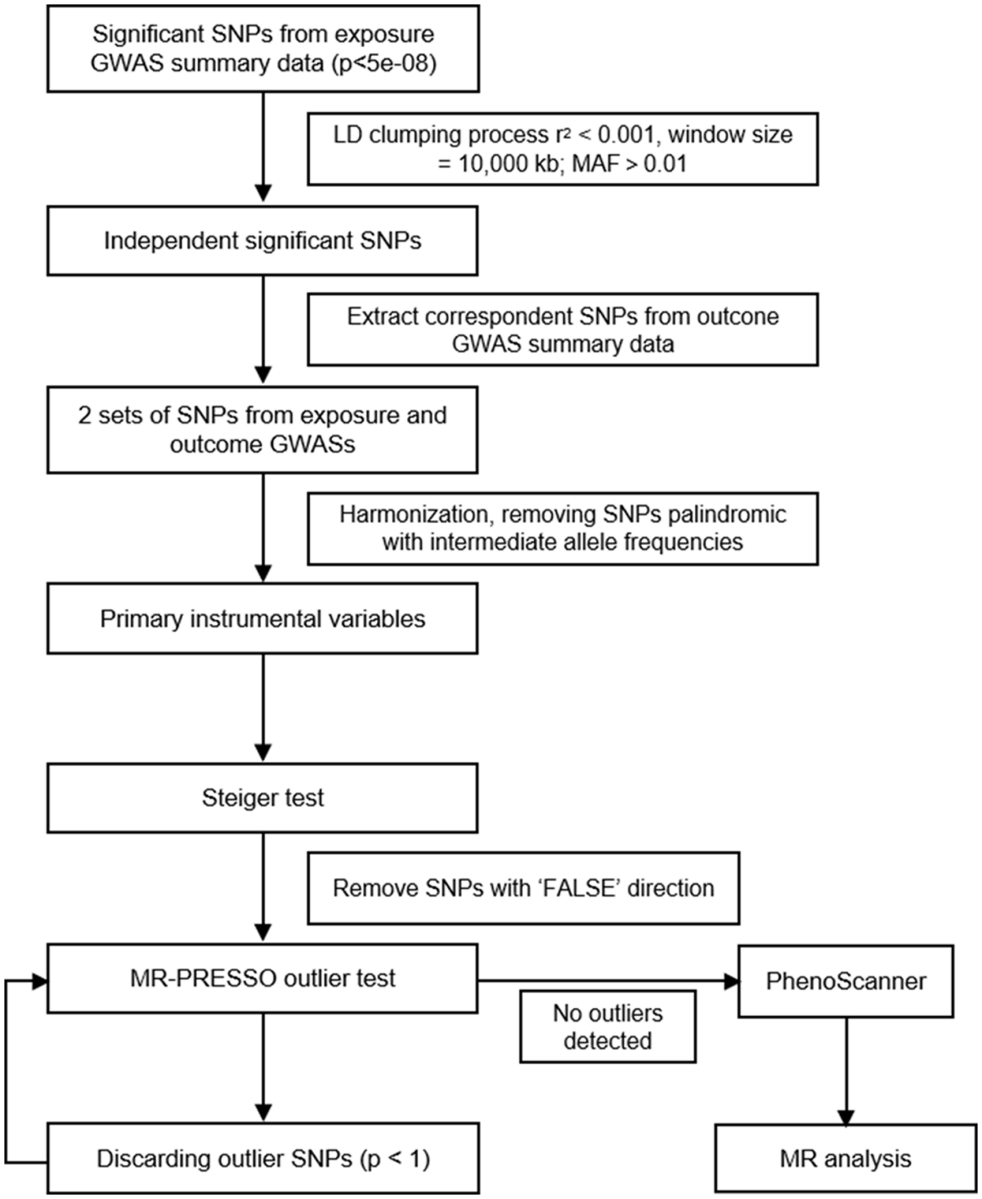
Flowchart of IVs selection strategy.

Valid IVs need to meet the following three assumptions: (1) association with the risk exposure of interest (relevance); (2) no shared common cause with the outcome (independence); and (3) they affect the outcome only through the risk factor (exclusion restriction assumption) (Davies et al., 2018). To ensure that all included IVs were valid, we employed a series of stringent control steps (Figure 1). First, we extracted genome-wide significant (*p* < 5 × 10^−8^) single-nucleotide polymorphisms (SNPs) from exposure GWASs. Since no genome-wide significant SNPs were detected in the GWAS of OCD, we chose to use suggestive significant SNPs (*p* < 1 × 10^−5^) as IVs. We then performed linkage disequilibrium (LD) clumping (R^2^ < 0.001, window size = 10,000 kb) based on the European 1,000 Genomes Project reference panel to select independent significant SNPs. Those with minor allele frequency (MAF) >0.01 and the lowest value of p were retained. F and weak instrumental variables.

Using the selected SNPs, we extracted SNPs from the outcome/PD GWASs. For those absent in the outcome GWASs, a proxy SNP in LD (*R*^2^ > 0.8) with the requested SNP was searched according to 1,000 genomes of European samples. After harmonisation of the two aforementioned sets of SNPs, palindromic SNPs with intermediate allele frequencies were removed, and the remaining SNPs were kept as primary IVs. Next, we performed the Steiger test for each SNP to determine whether the R^2^ (variance of disease/trait explained by selected SNPs) of the exposure was larger than the R^2^ of the outcome. SNPs tested as “FALSE” direction (*R*^2^ of the outcome >*R*^2^ of the exposure) would be excluded.

The horizontal pleiotropy, which occurs when an IV affects the outcome outside of its effect on the exposure, is the main factor that violates the assumptions (Hemani et al., 2018). The MR pleiotropy residual sum and outlier (MR-PRESSO) test was developed to identify horizontal pleiotropy in multi-instrument summary-level MR testing (Verbanck et al., 2018). It is a sequential method comprising three components: a global test for horizontal pleiotropy, an outlier test for each genetic variant, and a distortion test of the difference in the causal estimates before and after outlier removal. At the stage of IV selection, we adopted a stringent filtering step by discarding SNPs with a value of *p* < 1 in the outlier test and repeated this step until no outliers were detected. Finally, the remaining SNPs were uploaded to PhenoScanner to examine whether any of them was previously associated with PD (*p* < 5 × 10^−8^ in published PD GWASs) and remove possible confounding SNPs (Kamat et al., 2019).

Utilizing *R*^2^ (variance of exposure explained by the selected IVs) generated from the Steiger test, we calculated the overall F statistics (F = *R*^2^ [n – k – 1]/k[1 − *R*^2^], n: sample size, k: number of IVs) to quantify the strength of the IVs, and *F* > 10 was considered sufficient to overcome the weak instrumental variable bias (Burgess et al., 2013).

### MR analysis

The inverse variance weighted (IVW) fixed-effect method, which combines the ratio estimated using each variant in a fixed-effect meta-analysis mode, was adopted to estimate the causative effect of each exposure on the outcome (Burgess et al., 2013). Because the IVW method provides an unbiased causal estimation only when all IVs are valid, we adopted other methods of sensitive analysis when a violation of the assumptions inevitably existed. The weighted-median method can provide a consistent estimate when 50% of the IVs are invalid, at the cost of reduced statistical power (Bowden et al., 2016). MR-Egger regression allows all genetic variants to be invalid IVs under the Instrument Strength Independent of Direct Effect (InSIDE) assumption, which indicates that the pleiotropic effects of genetic variants on the outcome are direct (not through a confounder) (Bowden et al., 2015). The MR-Egger regression method also provides an intercept term for estimating the average pleiotropic effect across genetic variants. Even though we adopted stringent control steps, the MR-PRESSO method might still detect horizontal pleiotropy in the global test in the absence of detected outliers, which may be caused by a violation of the InSIDE assumption (Verbanck et al., 2018). Therefore, if pleiotropy was detected by the MR-PRESSO global test or the value of *p* of MR-Egger intercept was below 0.05, we used the results from the weighted-median method as the main method. The Cochran’s Q statistic was implemented to quantify heterogeneities, and a value of *p* < 0.05 was considered significant heterogeneity. We also performed a leave-one-out sensitivity analysis by sequential exclusion of each SNP at a time to determine whether particular variants were driving the association between the exposure and the outcome, and an IVW method was performed on the remaining SNPs. To ensure the stability of our results, we used another PD GWAS, conducted among the Finnish population, as the outcome as described above. Individual estimates were pooled using a fixed-effect meta-analysis. To adjust for multiple testing, a Bonferroni-corrected value of *p* was set as 0.05/8, meanwhile value of *p* < 0.05 was regarded as nominally significant.

In addition, we performed reverse MR to examine for reverse causality of PD on psychiatric disorders/traits. Genetic variant selection steps and MR analysis were conducted in a similar manner to that of forward MR. All statistical analyses were conducted using R version 4.2.1, with the use of the ‘TwoSampleMR’, ‘MRPRESSO’ and ‘META’ packages for the MR analysis.

## Results

Details of the IVs used for each MR analysis (both primary and validation analyses) are presented in the Supplementary Tables S1–S9. The main results, including causal estimation, the pleiotropy test, and the heterogeneity test, are presented in Tables 2, 3.

**Table 2:** Causal relations of psychiatric disorders with PD (IPDGC).

Exposure	Methods	nSNPs^a^	OR	95%CI	Pval	MR-PRESSO global Pval	Egger intercept Pval	Q_pval
AN versus PD	IVW^b^	6	1.172	(0.98, 1.40)	0.085	0.877		0.842
	MR Egger	6	1.327	(0.68, 2.58)	0.450		0.722	0.753
	Weighted median	6	1.150	(0.91, 1.46)	0.252			
Anxiety versus PD	IVW	3	0.895	(0.73, 1.10)	0.297	- ^d^		0.391
	MR Egger	3	1.540	(0.09, 26.29)	0.816		0.771	0.200
	Weighted median	3	0.872	(0.68, 1.12)	0.290			
BD versus PD	IVW	50	1.070	(0.98, 1.17)	0.136	0.443		0.502
	MR Egger	50	0.830	(0.53, 1.29)	0.411		0.254	0.515
	Weighted median	50	1.085	(0.95, 1.23)	0.215			
Insomnia versus PD	IVW	295	0.839	(0.58, 1.21)	0.348	0.081		0.068
	MR Egger	295	0.688	(0.18, 2.66)	0.588		0.857	0.063
	Weighted median	295	0.698	(0.42, 1.17)	0.175			
MDD versus PD	IVW	43	0.960	(0.77, 1.20)	0.723	0.491		0.488
	MR Egger	43	0.278	(0.09, 0.88)	0.035^c^		0.037^c^	0.651
	Weighted median	43	0.834	(0.59, 1.18)	0.305			
Neuroticism versus PD	IVW	79	0.997	(0.72, 1.38)	0.986	0.207		0.218
	MR Egger	79	0.543	(0.08, 3.94)	0.548		0.544	0.204
	Weighted median	79	1.133	(0.71, 1.81)	0.603			
OCD versus PD	IVW	20	0.994	(0.94, 1.05)	0.834	0.422		0.402
	MR Egger	20	1.025	(0.89, 1.19)	0.753		0.677	0.351
	Weighted median	20	0.973	(0.90, 1.05)	0.499			
Schizophrenia versus PD	IVW	141	1.042	(0.98, 1.12)	0.208	0.008^c^		0.008^c^
	MR Egger	141	0.920	(0.72, 1.18)	0.513		0.312	0.008^c^
	Weighted median	141	1.017	(0.93, 1.11)	0.702			

^a^nSNPs: number of SNPs used as instrumental variables; ^b^IVW: inverse-variance weighted; ^c^*p* < 0.05; ^d^Number of SNPs is not enough for MR-PRESSO pleiotropy test.

**Table 3:** Causal relations of psychiatric disorders with PD.

Outcome	Methods	nSNPs	OR	95%CI	Pval	MR-PRESSO global Pval	Egger intercept Pval	Q_pval
BD	IVW	19	1.059	(1.02, 1.09)	0.001^#^	0.741		0.725
	MR Egger	19	1.074	(0.99, 1.17)	0.124		0.586	0.671
	Weighted median	19	1.059	(1.01, 1.11)	0.014^*^			
BDI	IVW	16	1.074	(1.03, 1.12)	0.002^#^	0.946		0.732
	MR Egger	16	1.067	(0.95, 1.20)	0.297		0.568	0.694
	Weighted median	16	1.075	(1.01, 1.14)	0.019^*^			
BDII	IVW	20	1.025	(0.96, 1.10)	0.476	0.841		0.856
	MR Egger	20	1.072	(0.87, 1.32)	0.518		0.658	0.823
	Weighted median	20	1.038	(0.94, 1.14)	0.451			

^#^*p* < 6.25 × 10^−3^; *p < 0.05.

### Causal effect of psychiatric disorders on PD

For the primary analysis, instrumental variables from GWAS of psychiatric disorders collectively explained 0.18–4.40% of the variance. The *F*-statistic ranged from 22.40 to 109.40, suggesting no possible weak instrument bias. Under the IVW model, genetic predispositions to psychiatric disorders were not associated with PD (Table 2). Although we performed stringent control steps and no outliers were detected before we conducted MR analysis, the MR-PRESSO global test still indicated the existence of pleiotropy among IVs from schizophrenia GWAS, which indicated that the violation of the InSIDE assumption made causation estimates of IVW and MR-Egger unreliable. However, the estimates calculated using the weighted-median method were not significant. Horizontal pleiotropy in variants generated from MDD was detected by the MR-Egger regression intercept (*p* = 0.037), but not by the MR-PRESSO global test (*p* = 0.491). Therefore, it is more precise to adopt the estimation from MR-Egger calculation that genetically predicted MDD was negatively nominally associated with PD risk (OR = 0.278, 95%CI, 0.09–0.88, *p* = 0.035). However, this association was not confirmed by the weighted-median method. Leave-one-out analysis suggested no potential influence of a particular variant on the estimate (Supplementary Tables S2–S9).

In the validation analysis, no horizontal pleiotropy was detected using the MR-PRESSO global test, and the Cochran’s Q statistic value of *p* suggested no heterogeneity within each set of variants. Under the IVW model, genetically predicted AN was associated with PD risk (OR = 1.381, 95% CI = 1.02–1.87, *p* = 0.037). However, this association was not confirmed in the sensitivity analysis, which also showed a different association direction. Anxiety, insomnia, and MDD were predicted to be inversely associated with PD risk. Similar to AN, the causal estimation of anxiety in PD was not confirmed by other methods, which also showed a different association direction. However, estimation of the causal effect of insomnia and MDD on PD was validated by sensitivity analysis in the same direction, without observance of pleiotropy and heterogeneity. For neuroticism, the MR-Egger regression intercept indicated the presence of pleiotropy (*p* = 0.03), and MR-Egger showed an opposite causal estimate of PD risk (OR = 0.050, 95% CI = 0.00–0.62, *p* = 0.022). The sensitivity analysis also showed the same causal direction. Leave-one-out plots demonstrated that these associations were unlikely to be driven by extreme SNPs (Supplementary Tables S2–S9).

Due to the inconsistent results of the primary and validation analyses, we meta-analysed the results using a fixed-effect model. No significant results were detected, except for anxiety disorders (Pooled OR = 0.84, 95%CI, 0.71–0.99).

### Causal effect of PD on psychiatric disorders

**Figure 2:**
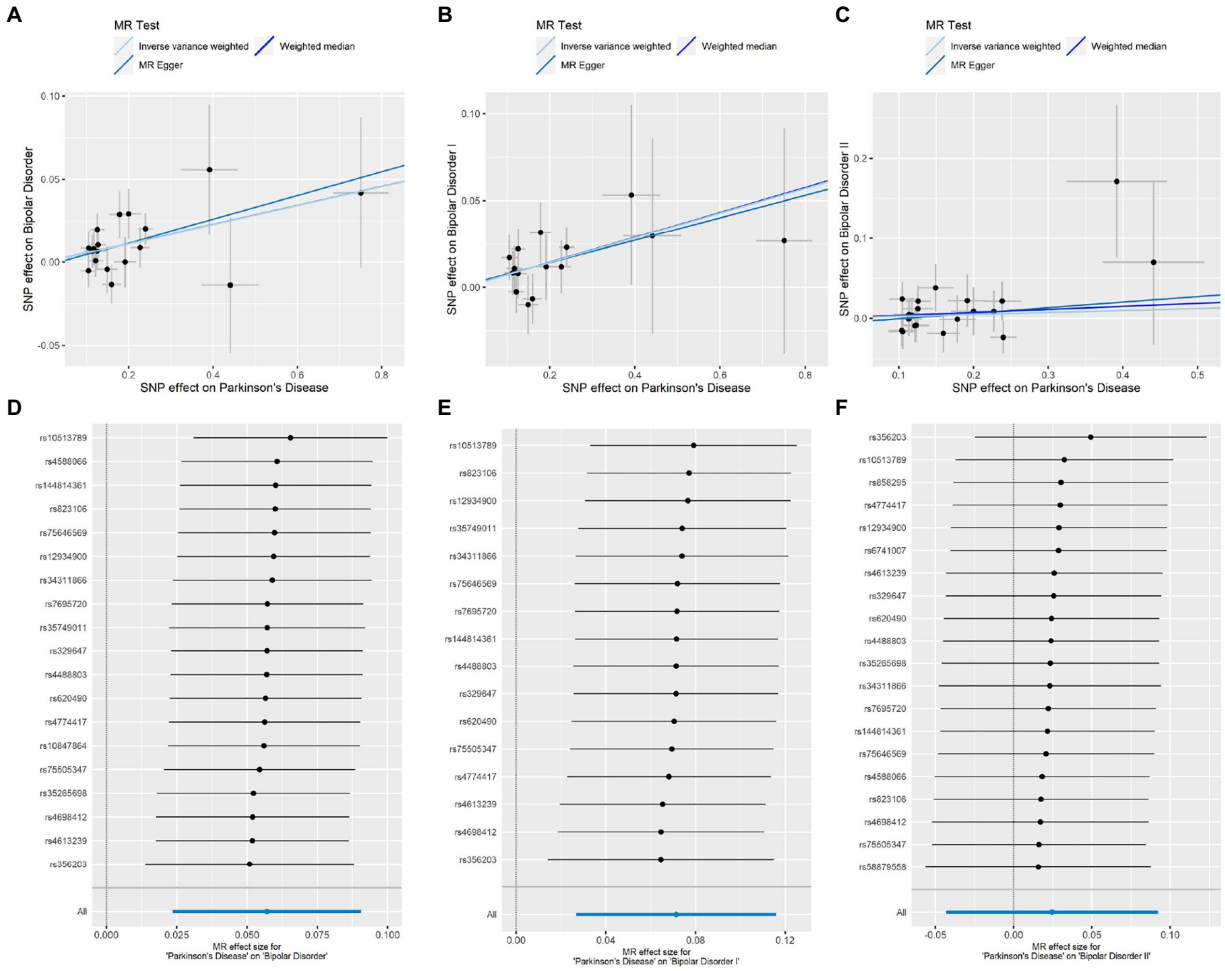
Causal effect estimation of PD on BD. **(A–C)** Scatterplots of potential effects of SNPs on BD (BD/BD I/BD II) and PD. Corresponding slope of each represents the estimated MR effect of each method. **(D–F)** Leave-one-out-sensitivity forest plots of PD-BD (BD/BD I/BD II) results.

To clarify the precise causal relationship between psychiatric disorders and PD, we performed a reverse MR analysis, with PD as the exposure and psychiatric disorders as the outcome. Interestingly, we detected a causal relationship between genetically predicted PD and BD risk (Table 3). No pleiotropy or heterogeneity was detected in these results, and leave-one-out analysis demonstrated no potentially influential SNPs driving the causal link (Figure 2).

## Discussion

Based on the potential relevance between psychiatric disorders and PD, our study appears to be the first research using bidirectional two-sample MR analysis to thoroughly investigate the causal relationship between genetically predicted psychiatric disorders/traits and PD. We found some evidence of different effects of psychiatric disorders/traits on the liabilities of PD. For MR detection of certain causal relationships, no reverse causal association was detected in the reverse MR analysis. Notably, we found evidence for the causality of PD on the risk of developing BD.

Although we found no statistical significance to support a causal association between AN and PD in the discovery cohort, the sensitivity analysis showed a consistent trend. Furthermore, a causal relationship was observed in the validation cohort. To our best knowledge, few studies have addressed the association between AN and PD. Although both diseases share some common clinical and pathological phenotypes, such as dopamine deficits, hyposmia, micrographia, and hypophonia (Roessner et al., 2005; Schreder et al., 2008; Sekar et al., 2010; Stievenard et al., 2017; Favier et al., 2020), the relationship between these diseases has yet to be firmly verified. Hence, this MR result needs to be interpreted cautiously and requires confirmation through additional relevant studies.

To date, many prospective historical cohorts and observational studies have proven epidemiological links between depression and PD (Wang et al., 2018). Many studies have attempted to demonstrate that depression and PD shared a similar pathophysiological brain dysfunction. Specifically, monoamine deficiency (i.e., dopamine, 5-hydroxytryptamine, and noradrenaline), one of the earliest suggested biological mechanisms of MDD, has also been observed in PD patients, particularly in early and prodromal patients with RBD (Hamon and Blier, 2013; Barber et al., 2018). In addition to monoaminergic neurotransmission and chronic inflammation, gamma-aminobutyric acid decline and cerebral atrophy are considered common pathophysiological characteristics underlying depression and PD (Gao and Bao, 2011; Saiki, 2014; Blaszczyk, 2016; Huang et al., 2016; D'Mello and Swain, 2017; Li et al., 2022). On one hand, since approximately 60% of PD patients have experienced psychosis, and many are taking or have taken antipsychotic medications (Weintraub et al., 2017), clinicians are more inclined to misdiagnose drug-induced parkinsonism as PD. On the other hand, as a frequently observed symptom of NMS, depression is more likely to be considered a common complication secondary to PD. First, in the discovery cohort, our analysis revealed a clear causal relationship between MDD and PD. This result was verified by subsequent duplicating assays with different GWAS. However, instead of increasing the risk of PD, genetically predicted MDD had a protective role against PD risk. Such opposing trends have also been observed in the association between insomnia and PD. However, the significant finding in the validation MR (IVW: OR = 0.563, 95%CI: 0.33–0.97, *p* = 0.037) was not supported by the sensitivity analysis. Moreover, these findings also contradicted those from the few current studies in this field, which considered that these diseases could predict an increased risk of PD (Weisskopf et al., 2003; Bower et al., 2010; Lin et al., 2015; Terracciano et al., 2021). This anomalous protective effect may be attributed to the survivor bias, caused by the reduced life expectancy of severe mental illness patients, and the increasing prevalence of PD in the older age group (Lees et al., 2009; Boef et al., 2015; Vansteelandt et al., 2018; Plana-Ripoll et al., 2019).

To clarify the exact causal relationship between psychiatric disorders and PD, we performed a reverse MR analysis, with PD as the exposure and psychiatric disorders as the outcomes. Interestingly, we detected a causal relationship between genetically predicted PD and BD risk. According to the Diagnostic and Statistical Manual Disorders, Fifth Edition (DSM-5), BD I and BD II are the major two clinical subtypes of BD, which are characterized by the classification of mania or hypomania. BD II is sometimes considered a milder form of BD I, because these two subtypes of BD share some phenomenological features (Liu et al., 2022). However, with increasing advances in BD epidemiology, clinical presentation (Zimmerman et al., 2013), and genetic basis (Charney et al., 2017), the important difference between the two subtypes is gradually being recognised. Further exploration of the specific role of BD subtypes in this association revealed that PD might be a risk factor for BD I rather than BD II. There is growing evidence that PD patients have a high prevalence rate of various psychiatric disorders. BD is generally considered a specific risk factor for PD, rather than an ensuing complication (Huang et al., 2019), which is contrary to our results. However, the diagnosis of PD is currently based on clinical manifestations which became evident when 50% of dopaminergic neurons were lost in the substantia nigra (Marsden, 1990). So it’s difficult to find out the exact beginning of the PD pathologic process. Taking the shared molecular mechanism into account, some may think it more appropriate that BD be viewed as an early symptom of PD (Pontone and Koch, 2019). As PD advances psychiatric complications emerge. 17% of patients with PD treated with dopaminergic therapy and 4% undergone DBS develop mania or hypomania, symptomatically similar to that of BD (Temel et al., 2006; Maier et al., 2014). In clinical terms, depression is not only a typical presentation of BD (Vieta et al., 2018), but also an important NMS of PD, which may hint to potential common clinical features between these diseases. However, similar to previous analyses, further related studies are needed to verify this finding.

Regarding other psychiatric disorders/traits, including OCD and schizophrenia, contrary to epidemiologic and other studies, we did not detect the causal association between these psychiatric conditions and PD. However, a close relationship exists between these two conditions. In the most updated nationwide longitudinal study published in 2022, OCD was reportedly an independent risk factor for PD (Liou et al., 2022). Another retrospective record-based case–control study demonstrated that schizophrenia spectrum disorder contributed to the incremental risk of PD in the elderly population (Kuusimaki et al., 2021). Understanding these potential association mechanisms may advance our knowledge of the underlying biology of these diseases and facilitate the early diagnosis of PD.

Our results should be viewed in the context of several limitations. Considering the existence of ethnicity-specific genomic heterogeneity, our findings must be interpreted with caution and may not be applicable to other racial/ethnic backgrounds, since all GWASs were conducted in European populations. Moreover, despite recent advances, existing GWAS methodology may still lack the ability to detect sufficient heritability of diseases, thus limiting the statistical power of the IVs used in this MR analysis. Owing to the lack of explicit measures and knowledge of potential confounders, pleiotropy may not be completely ruled out, and it is difficult to discuss the extent by which the results are influenced by this phenomenon. It is also difficult to determine the degree of sample overlap between the original GWASs of exposures and outcomes, which could cause weak instrument bias and violation of the independence assumption. However, bias from weak instruments in very large consortia may not be substantial and the F statistics was calculated to insure and minimize such bias (Burgess et al., 2016). For the violation of independence assumption, 2-sample MR methods can be safely applied to one-sample MR performed within large bio banks (Minelli et al., 2021). As presented above, many patients with psychiatric disorders have a history of antipsychotic use, which makes them more inclined to be diagnosed with drug-induced parkinsonism rather than PD. This possible misdiagnosis may have increased the effect of confounding factors on our results. Nevertheless, this study has several strengths. First, for each psychiatric exposure trait, we selected the latest and largest GWASs in European populations to ensure a reliable conclusion. Second, we applied a very stringent SNP quality threshold to reduce potential pleiotropic effects as much as possible. Moreover, this bidirectional MR analysis covered a wide variety of psychiatric disorders and traits, filling a gap in the observational literature and broadening our understanding of the relationship between psychosis and PD.

Collectively, in European populations, this 2-sample MR analysis provided evidence that genetically predicted psychiatric disorders may play various roles in the risk of PD. Concurrently, reverse MR analysis suggested that PD should be considered as a risk factor for BD subtype I. This significant result, supported by sensitive MR methods, showed no obvious heterogeneity or pleiotropy. Although these findings could be biased due to horizontal pleiotropy, we did not consider and detect hypotheses between psychiatric disorders and PD. As such, this topic merits further exploration.

## Data availability statement

The original contributions presented in the study are included in the article/Supplementary material, further inquiries can be directed to the corresponding author/s.

## Ethics statement

The studies involving human participants were reviewed and approved by The Ethics Committee of Xiangya Hospital of Central South University in China, the number of the approval is 202103191. The patients/participants provided their written informed consent to participate in this study.

## Author contributions

QW and SL performed the majority of the analyses and wrote the manuscript. SL and YX conducted the systematic review. JG designed the study. All authors contributed to the article and approved the submitted version.

## Funding

This study was supported by the national key plan for scientific research and development of China (2021YFC2501204), Technology Major Project of Hunan Provincial Science and Technology Department (grant no. 2021SK1010), the National Natural Science Foundation of China (grant no. 81873785, 82071439, 81974202, and U20A20355), Hunan Province Innovative Construction Project Science (grant no. 2019SK2335), and the Innovation-driven Team Project from Central South University (grant no. 2020CX016), the innovative team program from Department of Science & Technology of Hunan Province (grant no. 2019RS1010).

## Conflict of interest

The authors declare that the research was conducted in the absence of any commercial or financial relationships that could be construed as a potential conflict of interest.

The reviewer ZH declared a shared affiliation with the authors to the handling editor at the time of review.

## Publisher’s note

All claims expressed in this article are solely those of the authors and do not necessarily represent those of their affiliated organizations, or those of the publisher, the editors and the reviewers. Any product that may be evaluated in this article, or claim that may be made by its manufacturer, is not guaranteed or endorsed by the publisher.
