# Supplementary material for: Bidirectional Mendelian randomization study of psychiatric disorders and Parkinson’s disease
Source: Front Aging Neurosci. 2023 Mar 14;15:1120615. doi: 10.3389/fnagi.2023.1120615 (PMC10045982; doi:10.3389/fnagi.2023.1120615)

**Part 2. Supplement Figures: funnel plots, scatter plots and leave-one-out plots for relationship of genetically predicted psychiatric disorders with PD**

## Supplement Fig.1 Anorexia Nervosa VS PD

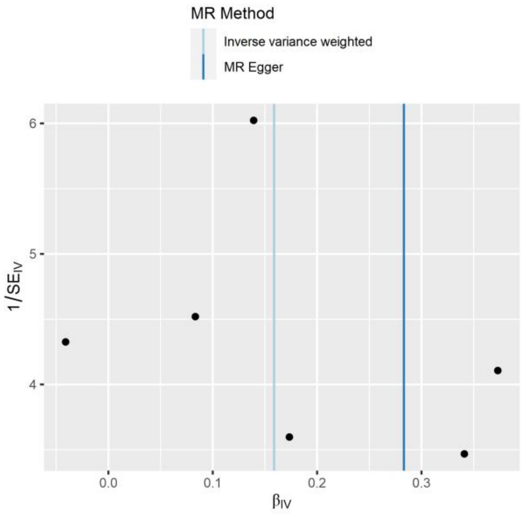


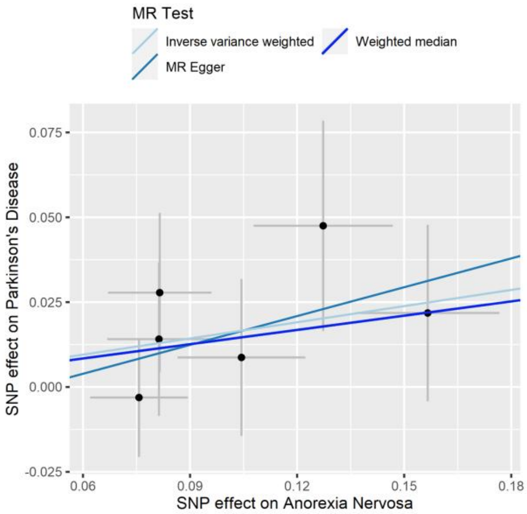


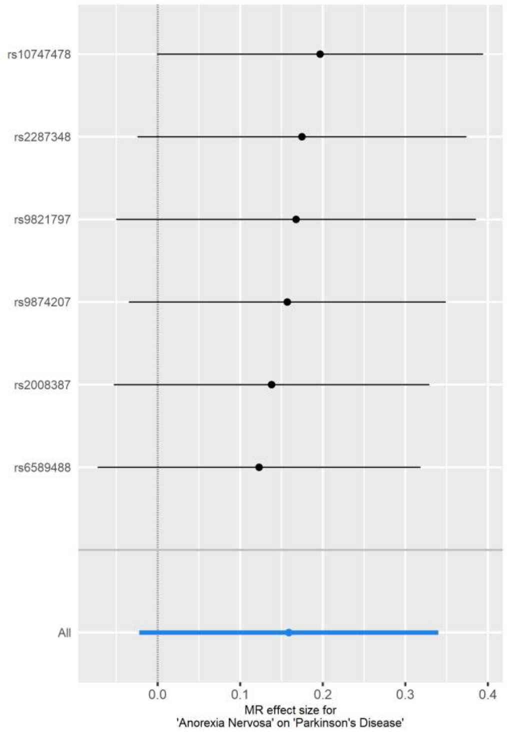

**Supplement Fig.2 Anxiety VS PD**

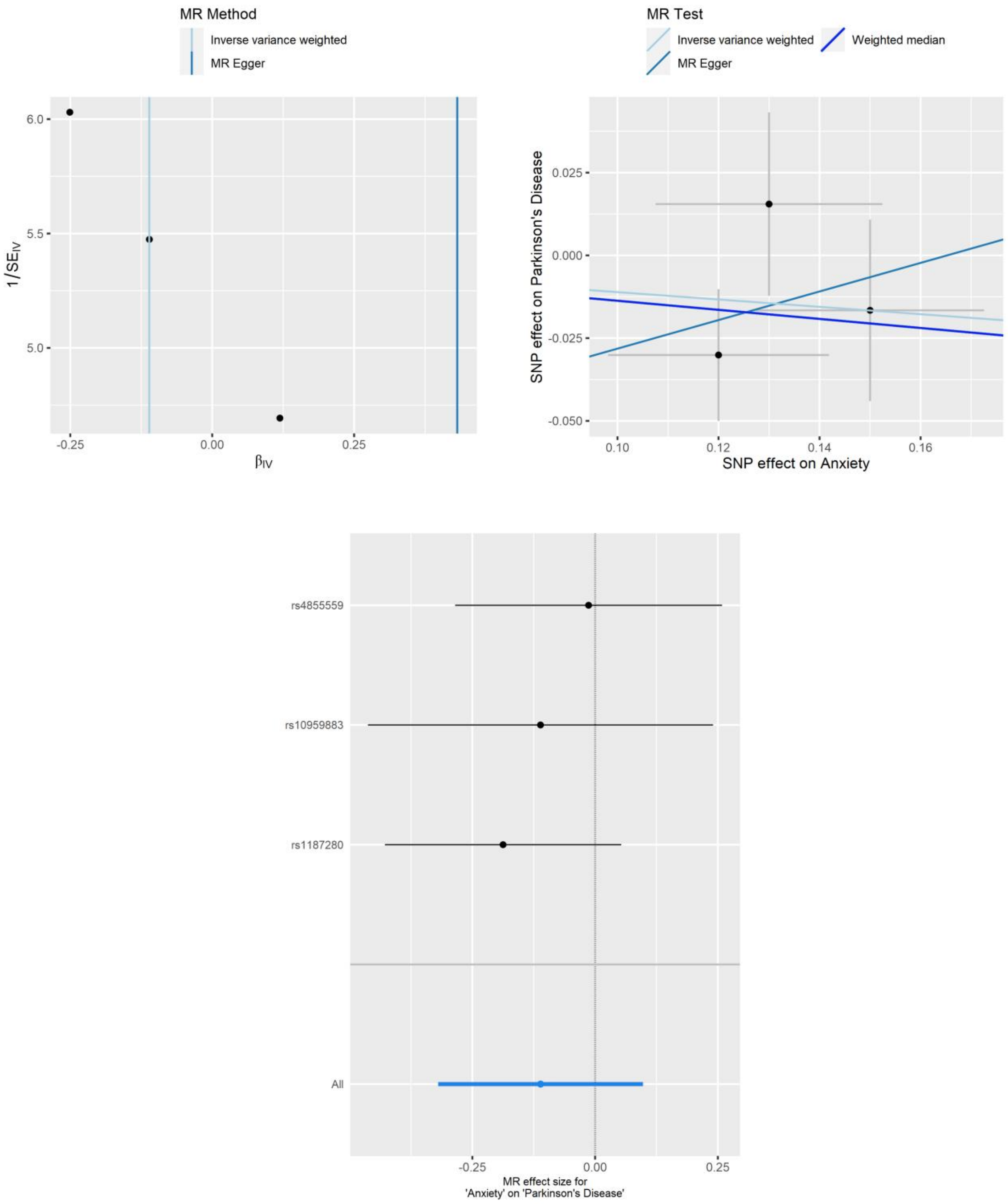

# Supplement Fig.3 Bipolar Disorder VS PD

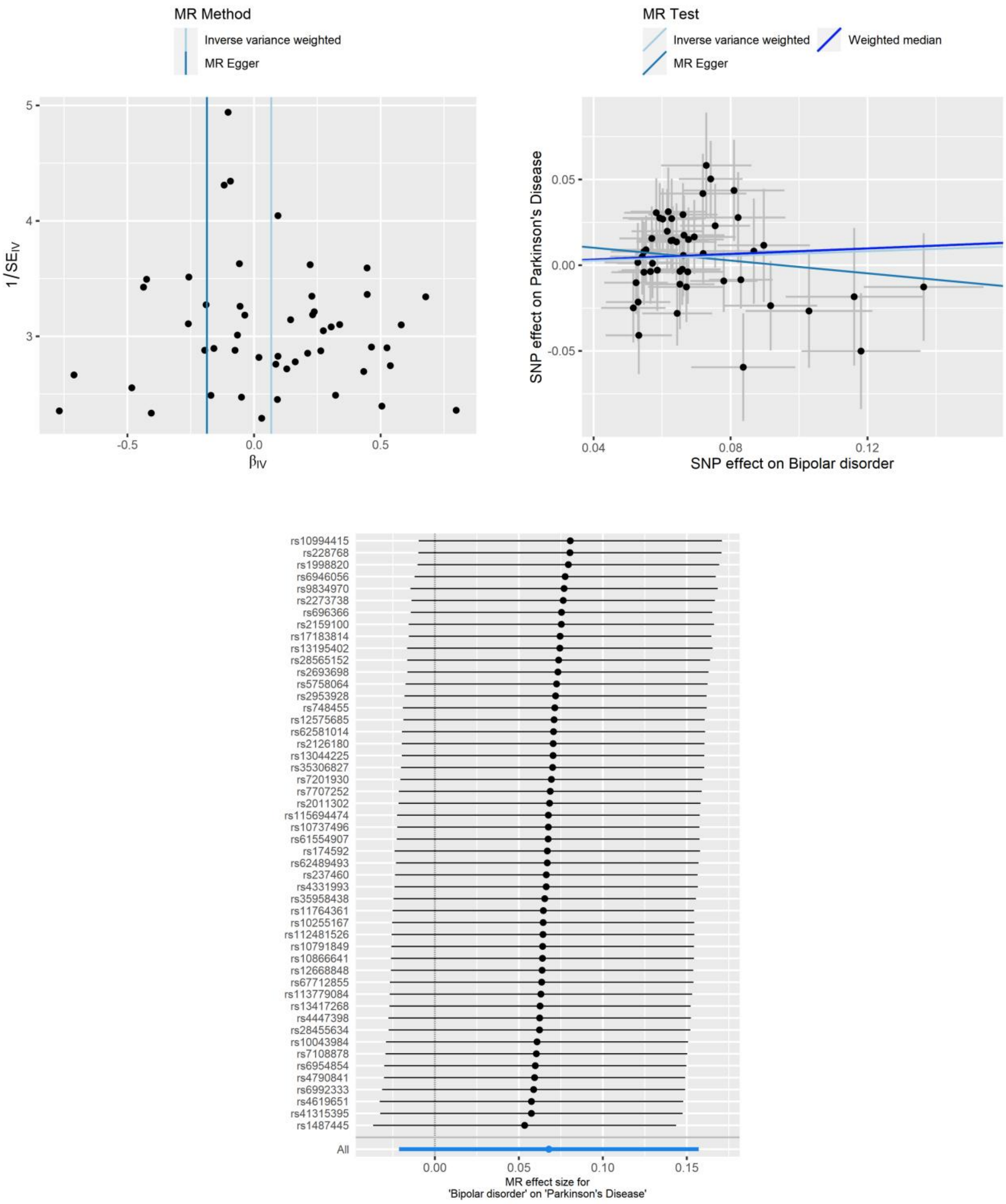

**Supplement Fig.4 Insomnia VS PD**

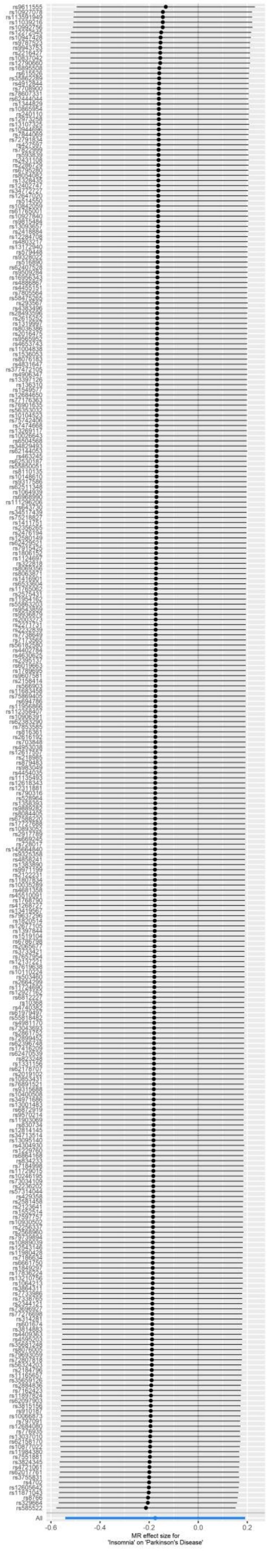


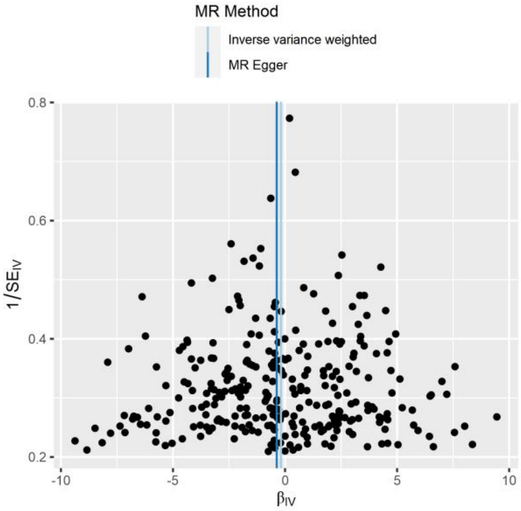


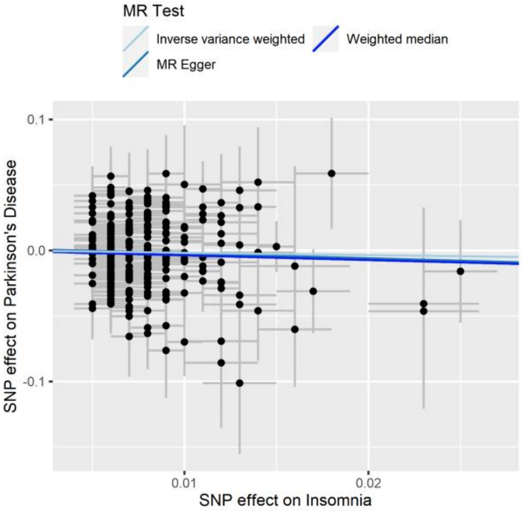

# Supplement Fig.5 MDD VS PD

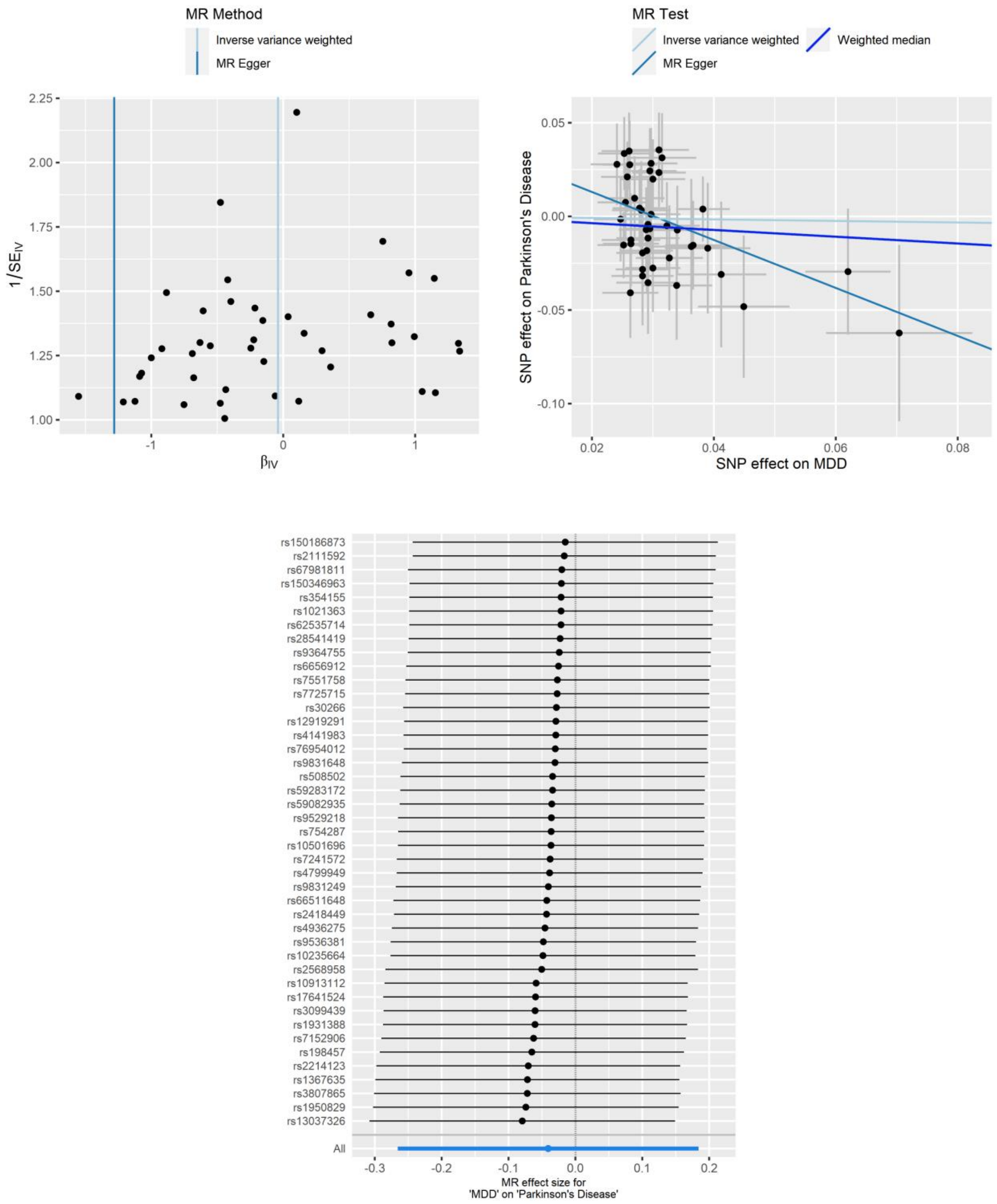

**Supplement Fig.6 Neuroticism VS PD**

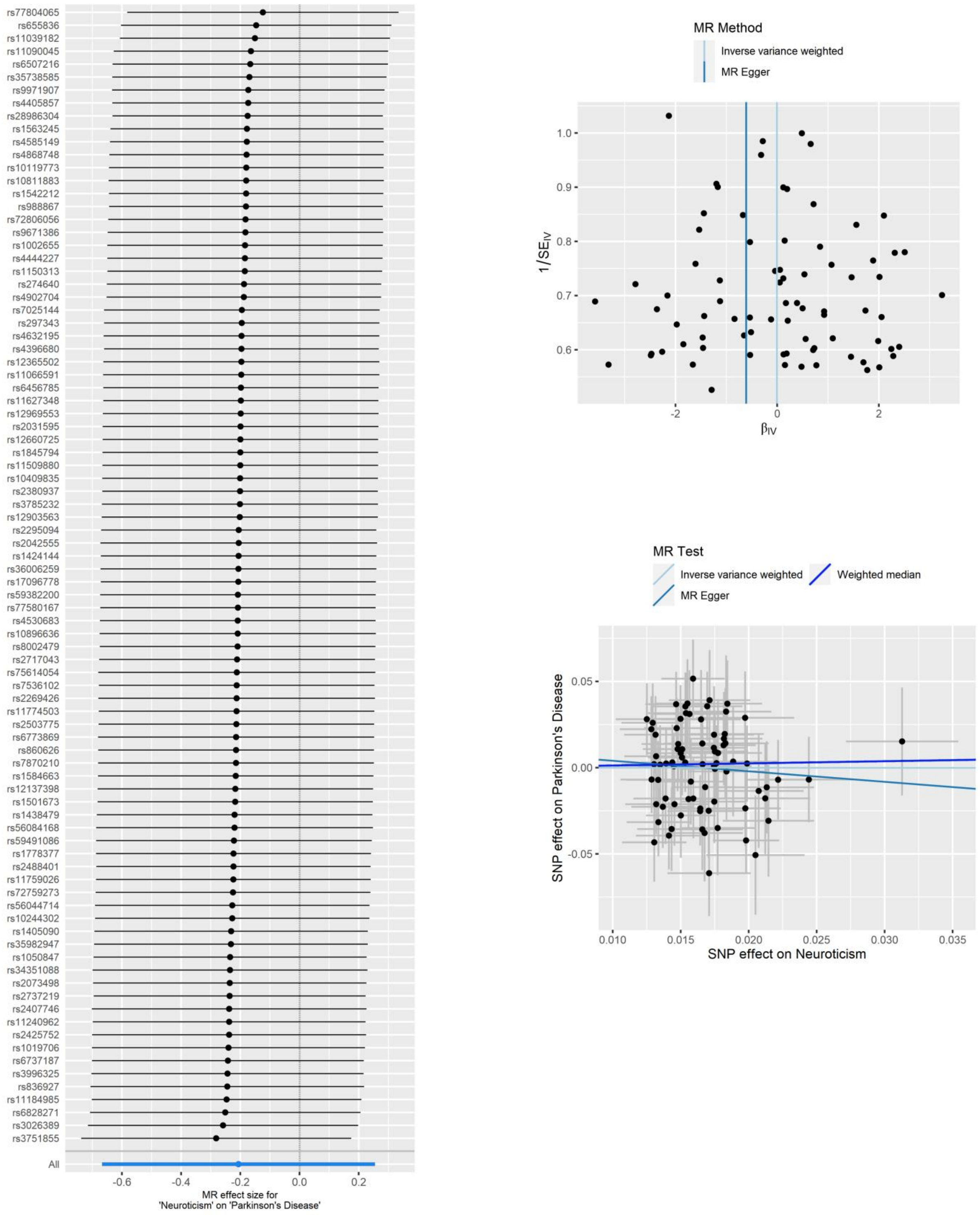

# Supplement Fig.7 OCD VS PD

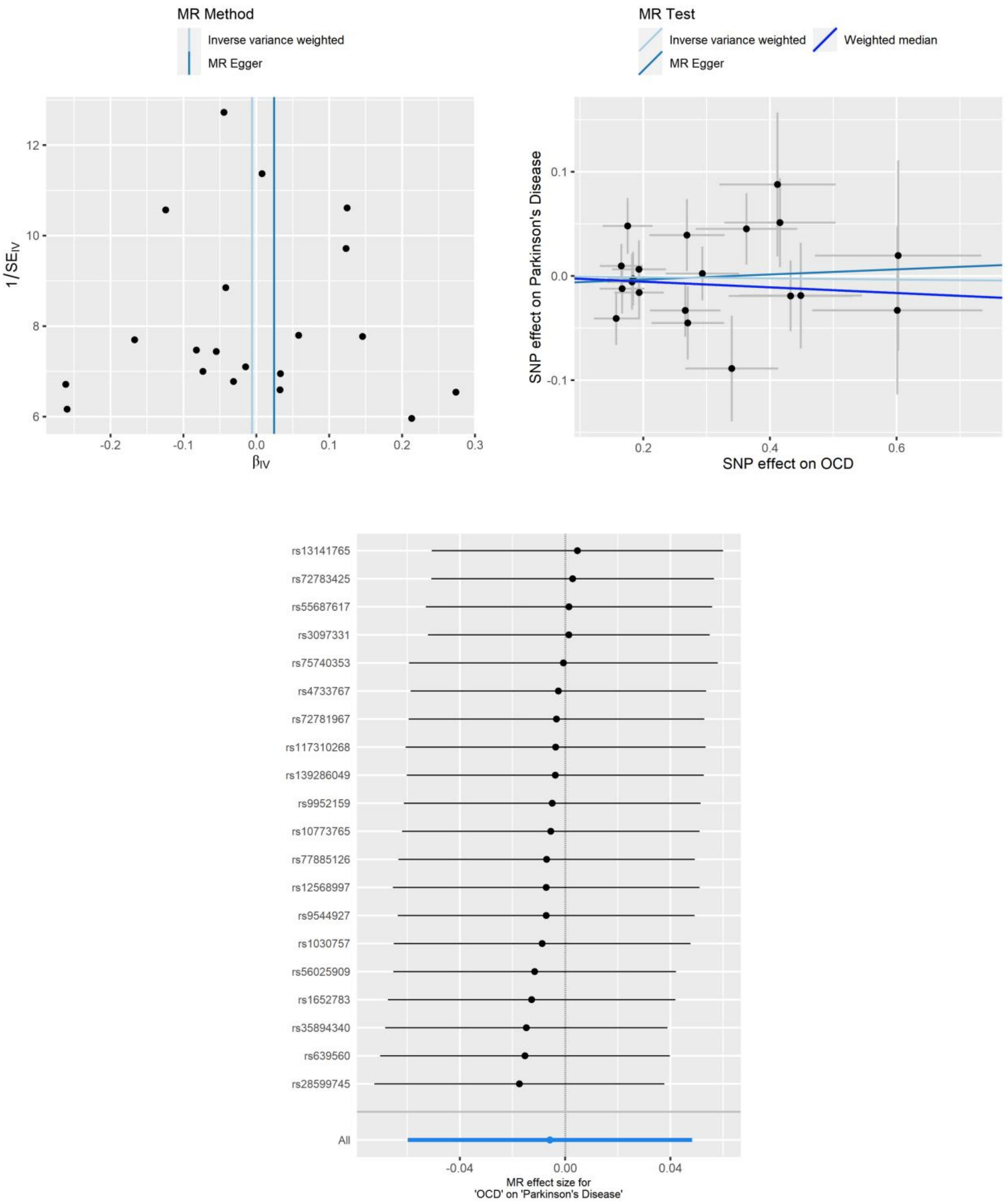

## Supplement Fig.8 Schizophrenia VS PD

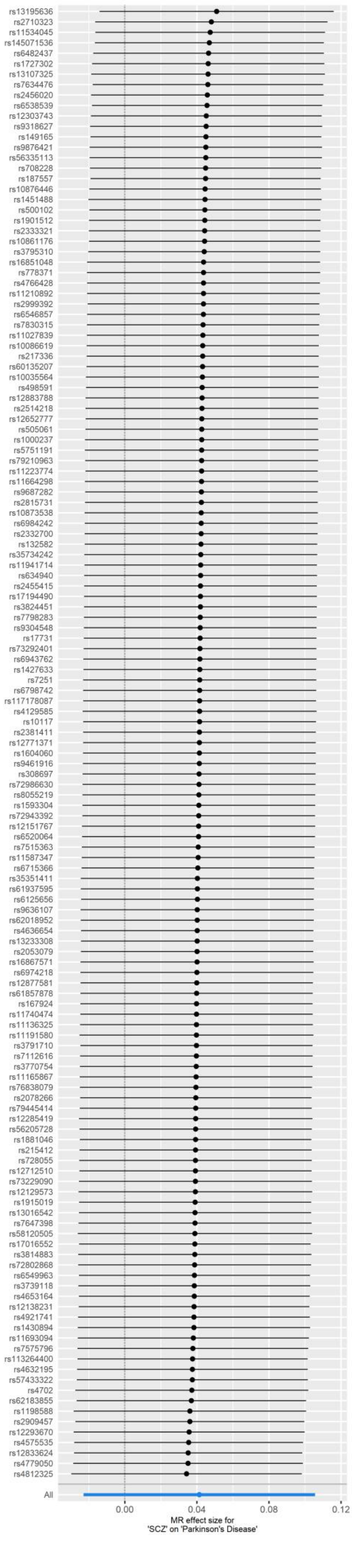


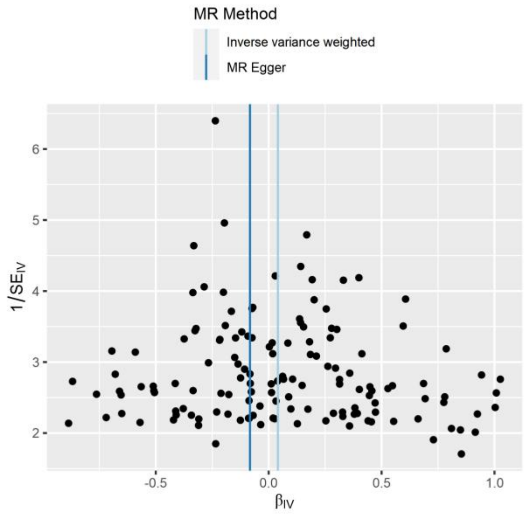


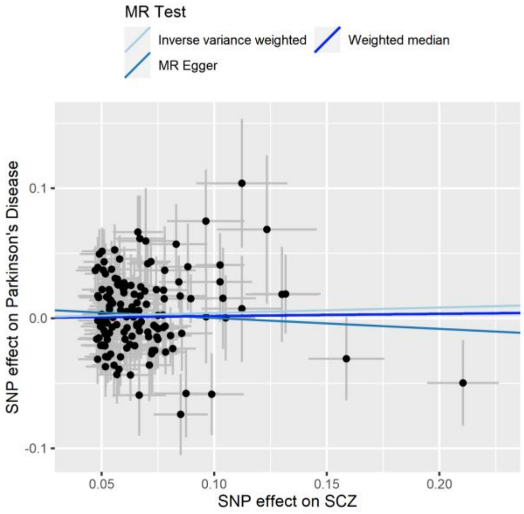

**Supplement Fig.9 Anorexia Nervosa VS PD (validation)**

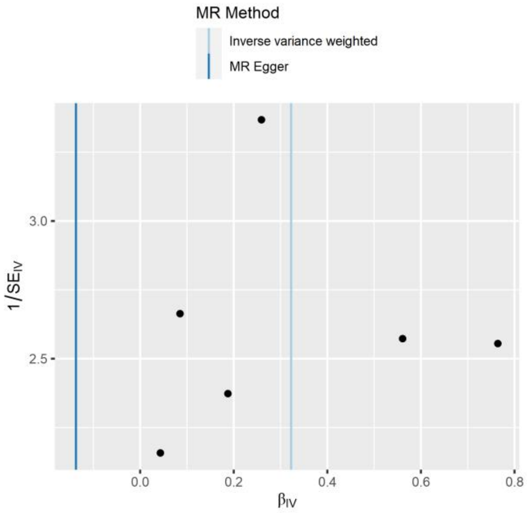


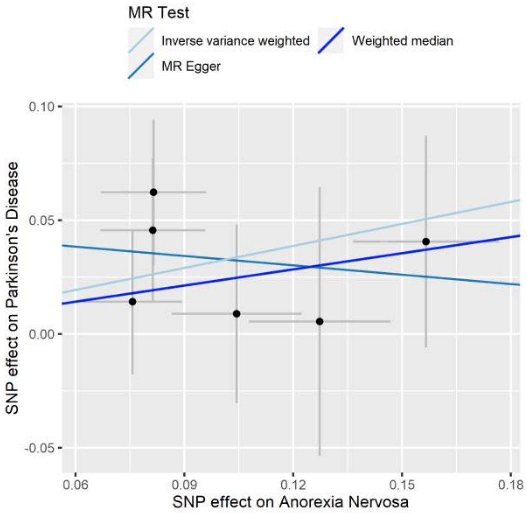


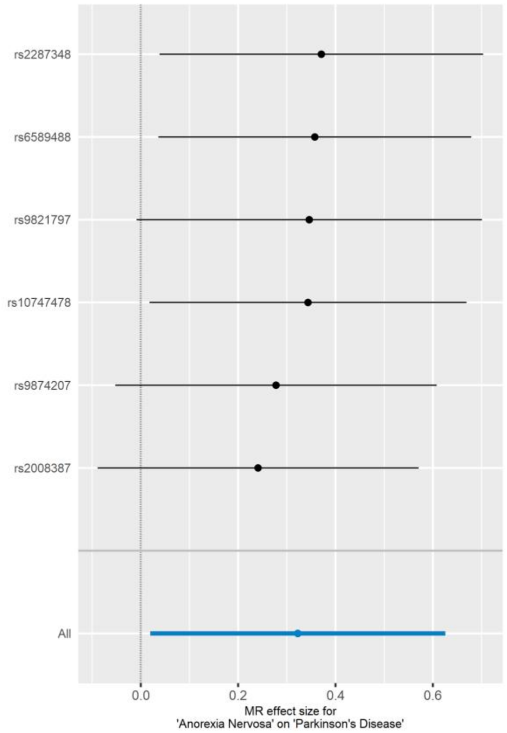

**Supplement Fig.10 Anxiety VS PD (validation)**

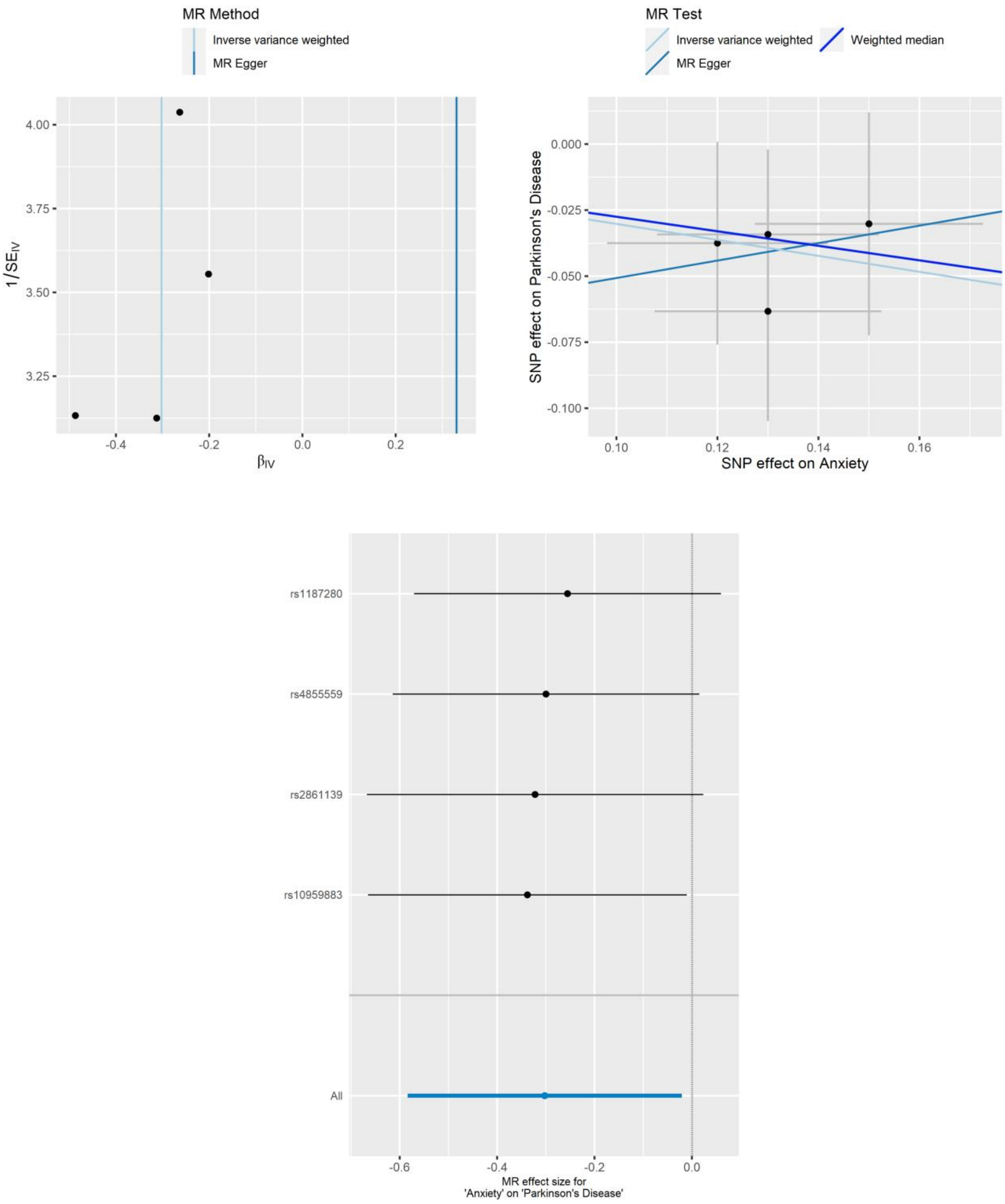

**Supplement Fig.11 Bipolar Disorder VS PD (validation)**

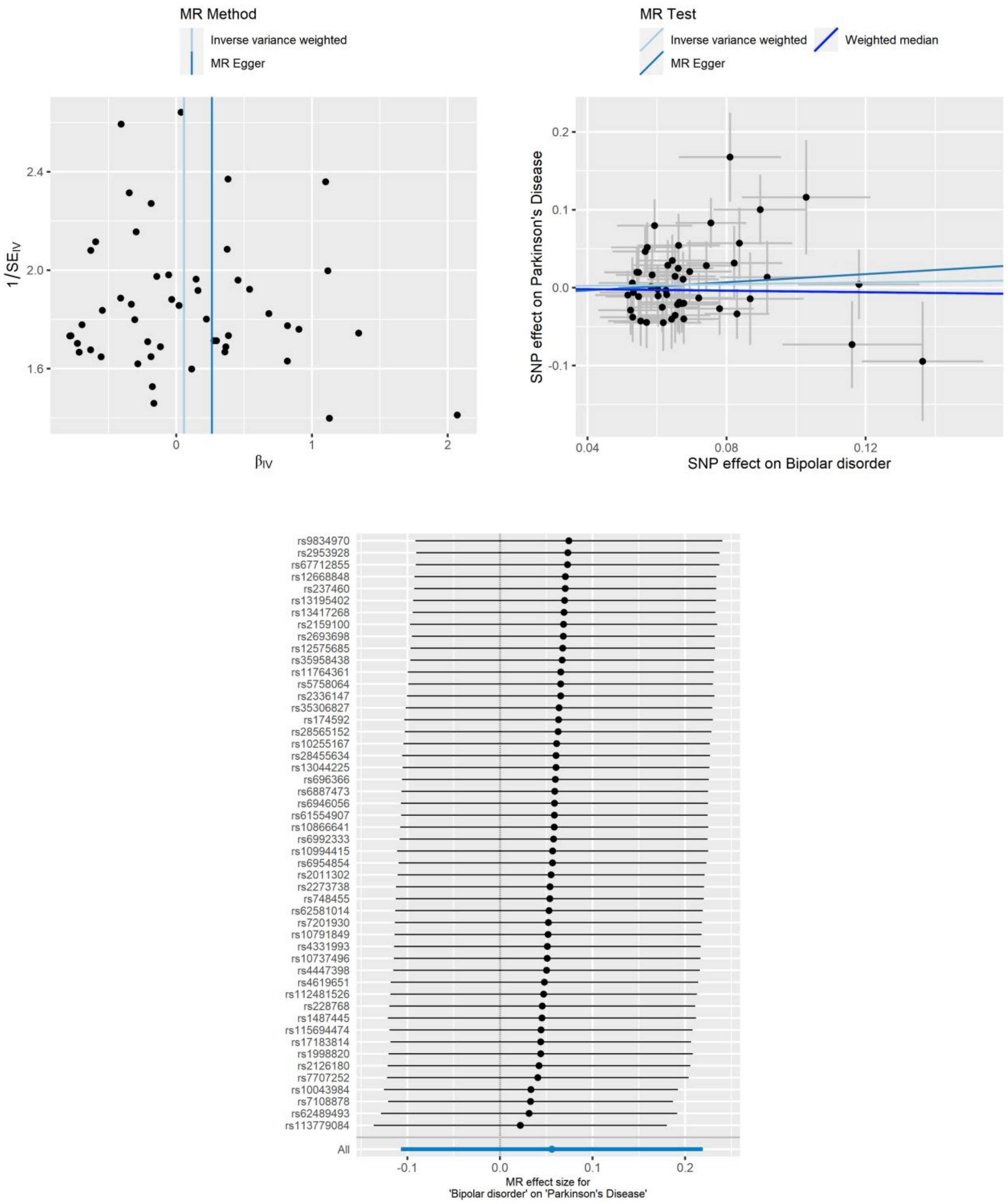

**Supplement Fig.12 Insomnia VS PD (validation)**

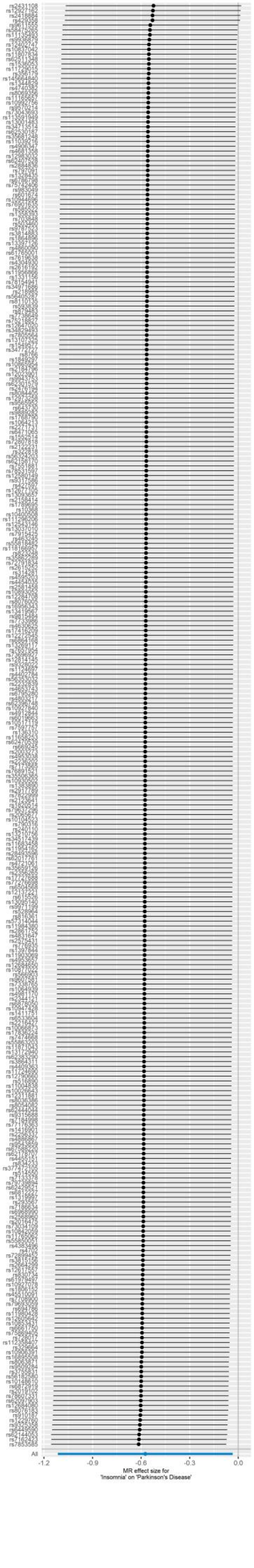


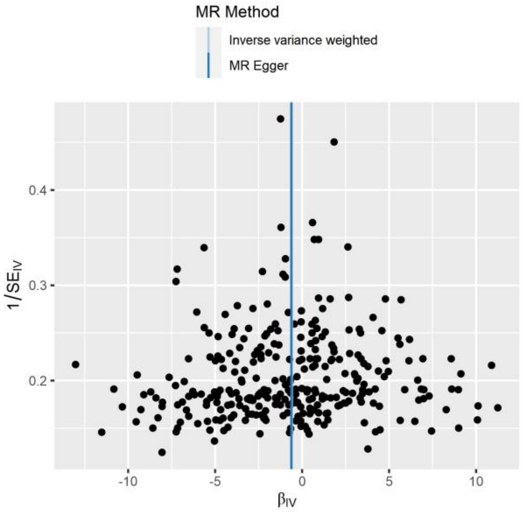


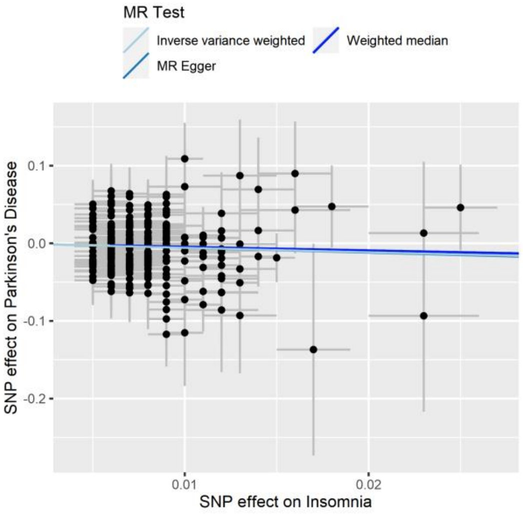

## Supplement Fig.13 MDD VS PD (validation)

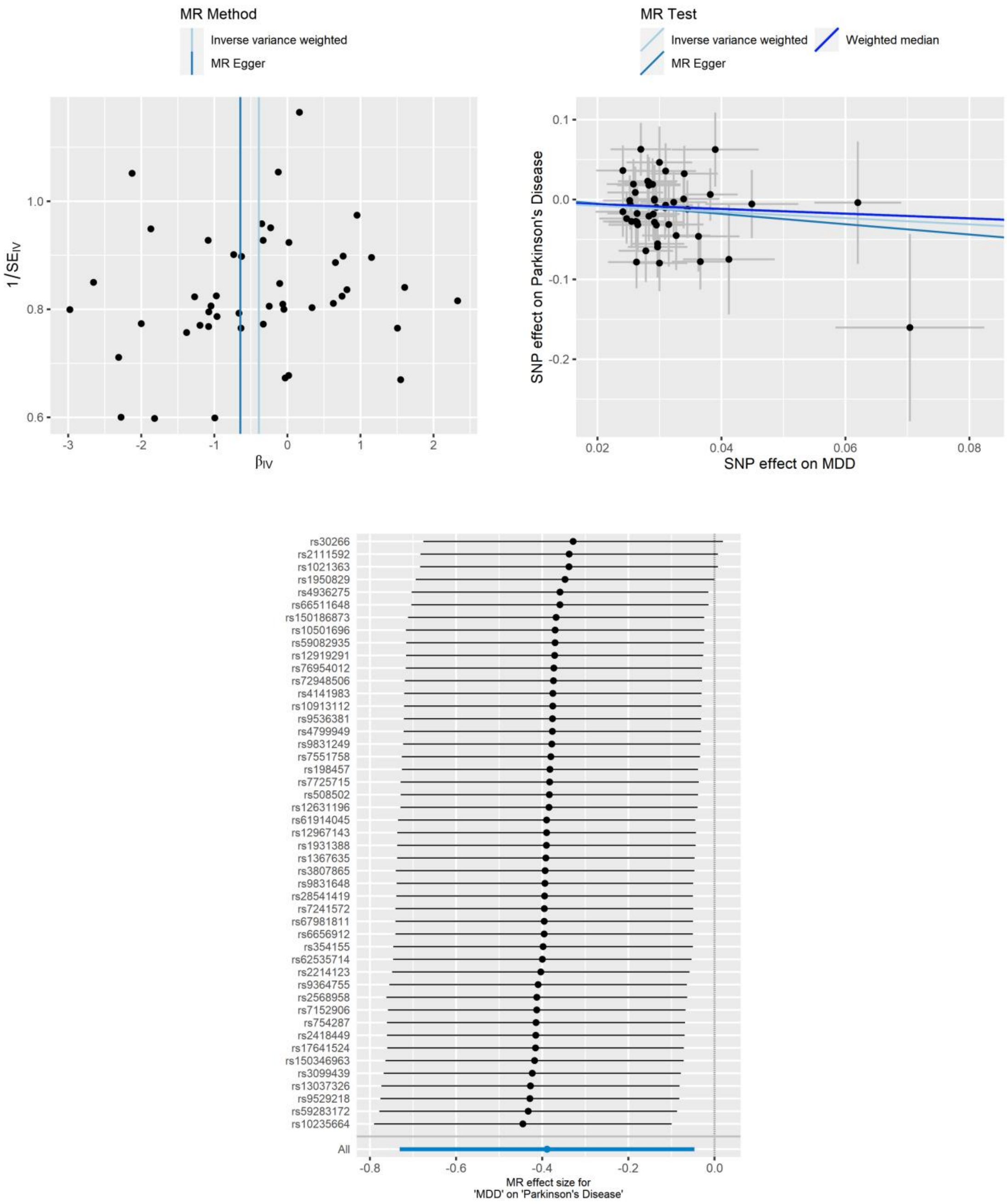

# Supplement Fig.14 Neuroticism VS PD (validation)

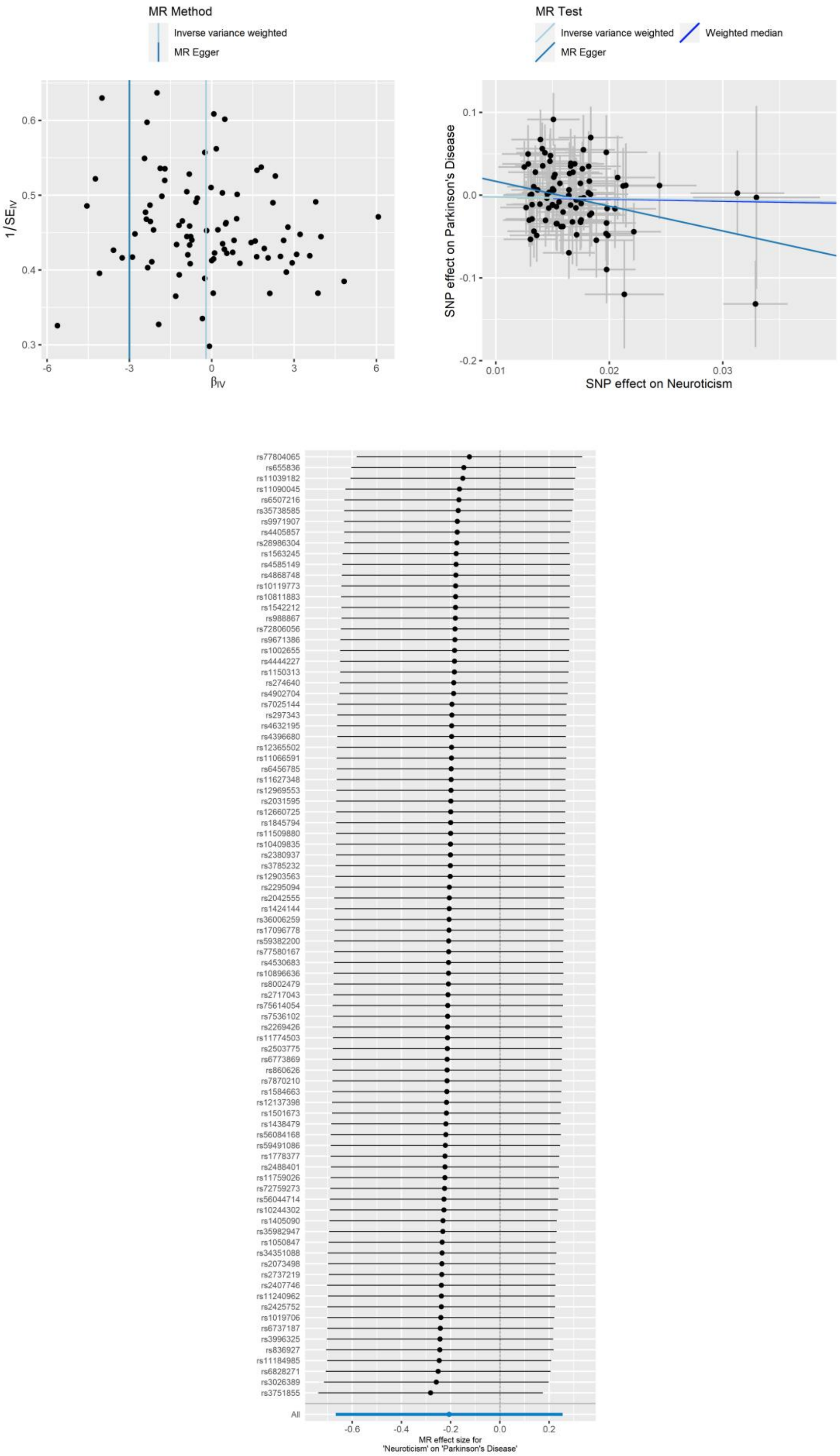

**Supplement Fig.15 OCD VS PD (validation)**

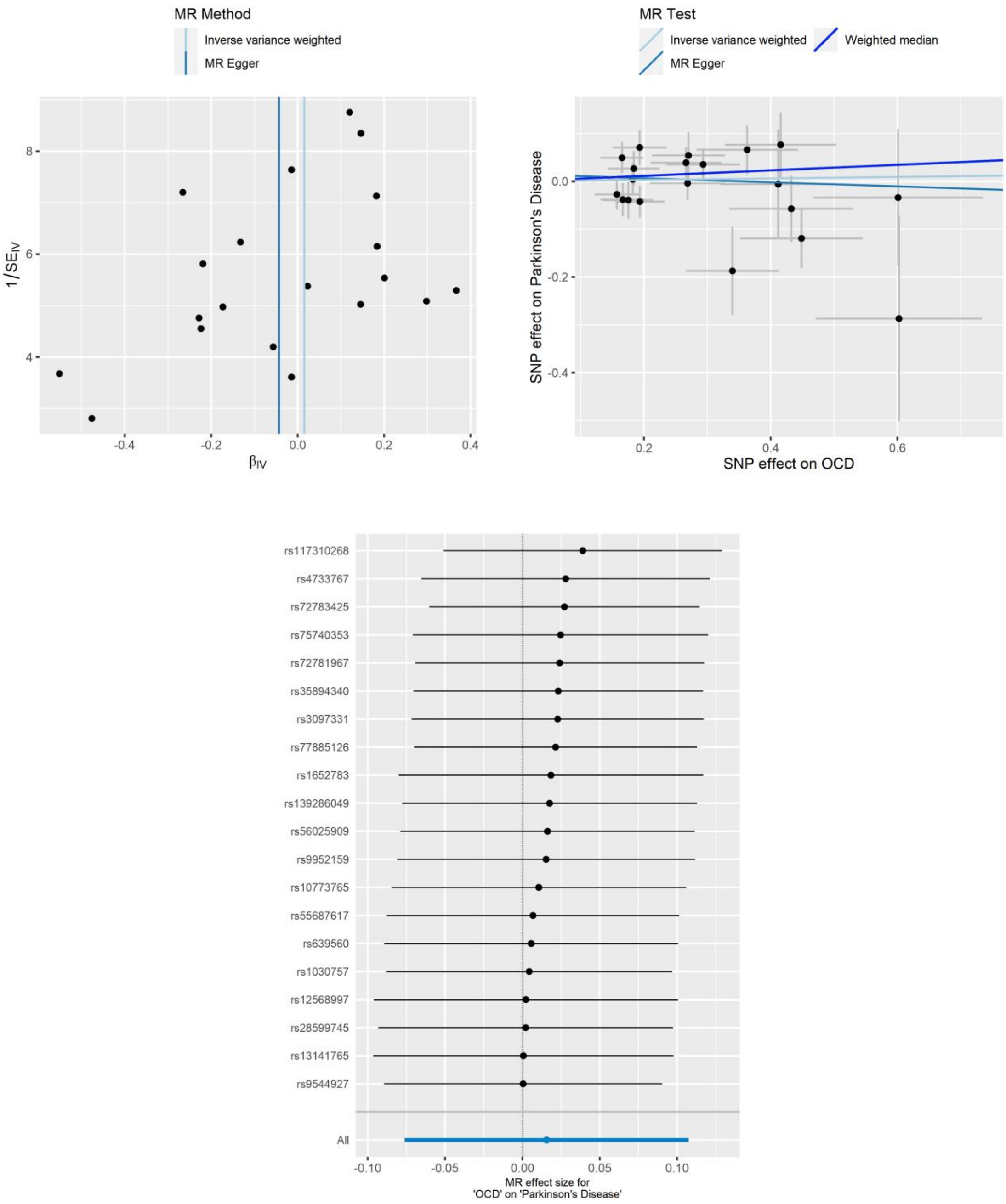

# Supplement Fig.16 Schizophrenia (validation) VS PD (validation)

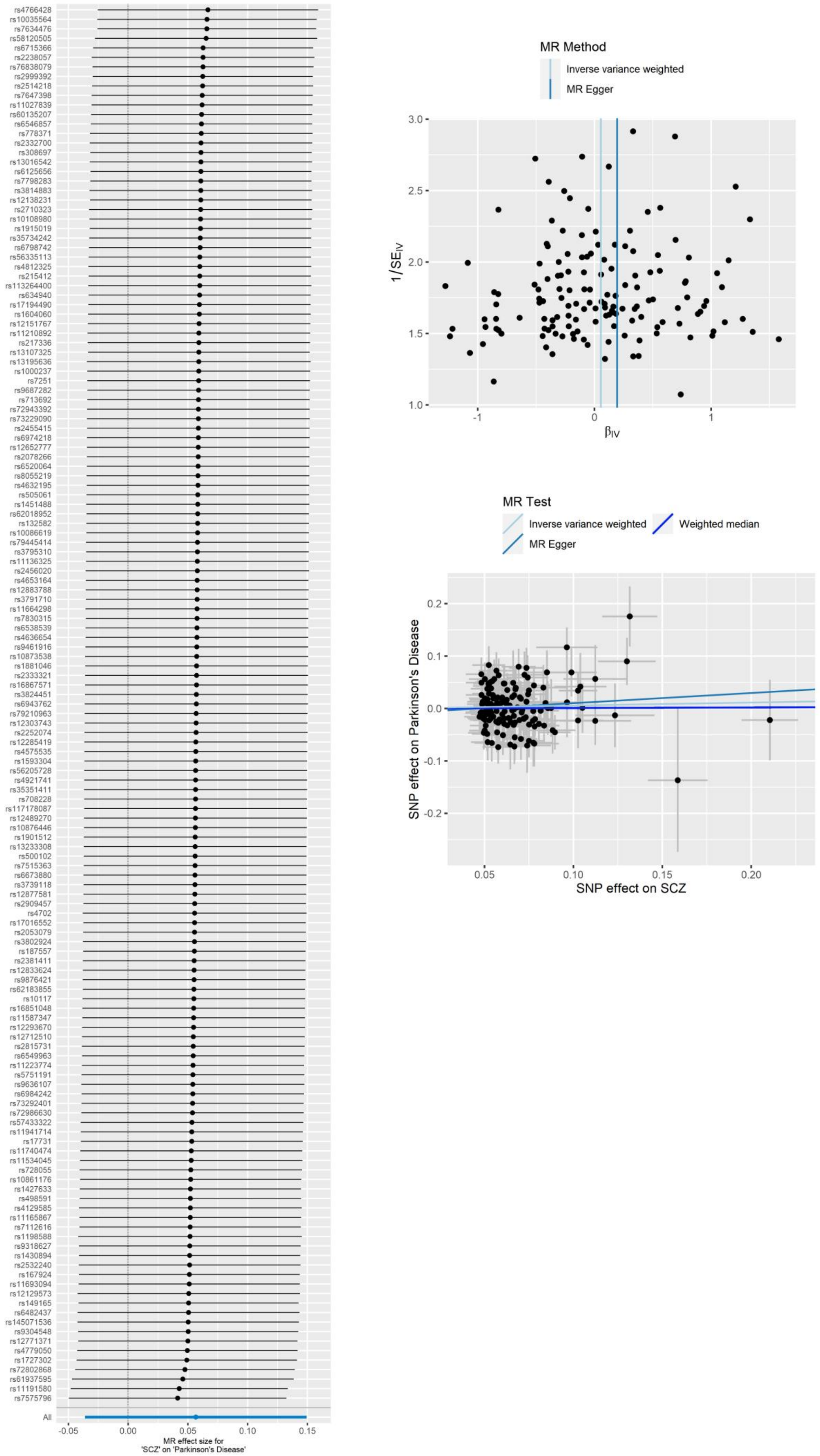

# Supplement Fig.17 PD VS Anorexia Nervosa

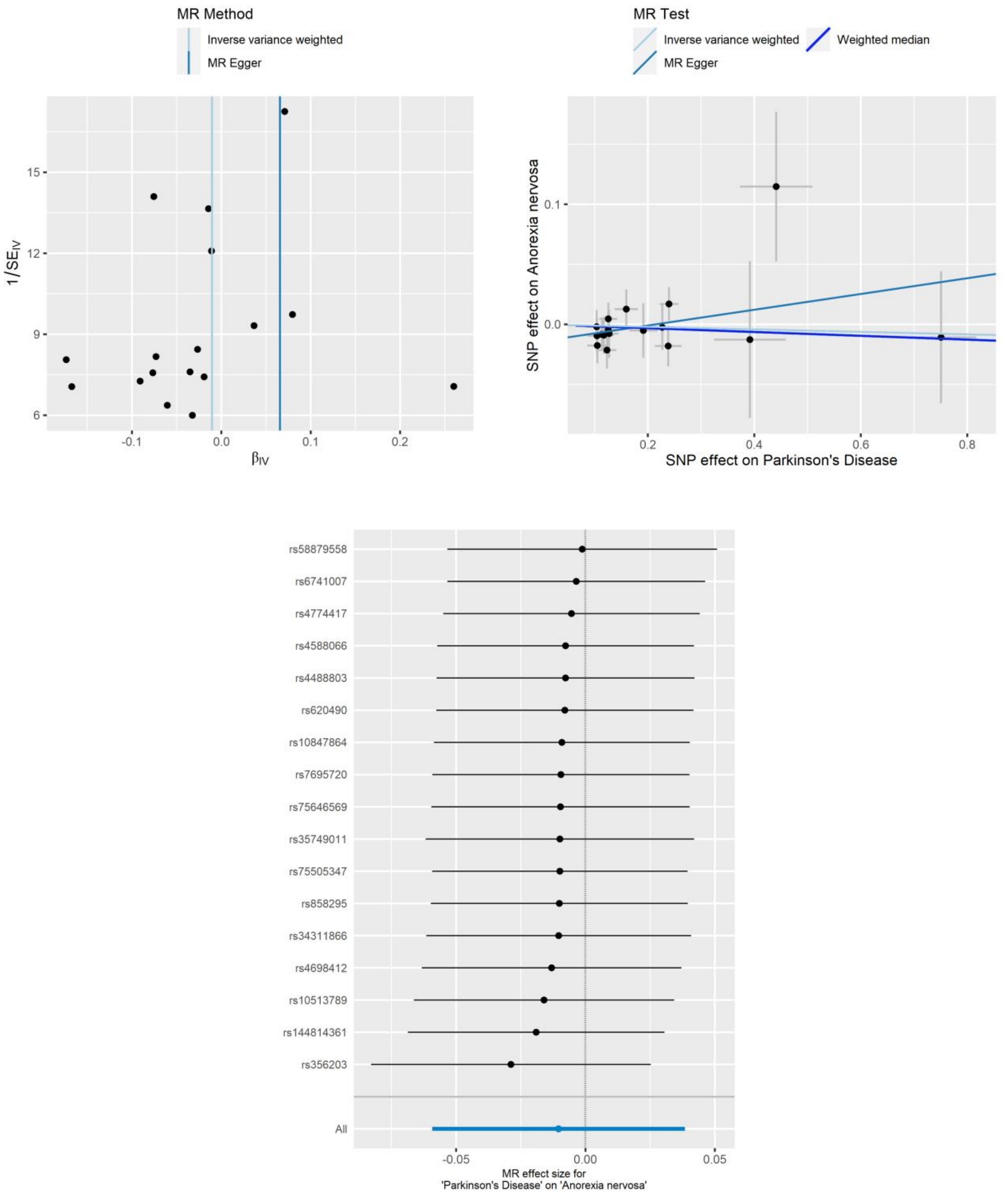

**Supplement Fig.18 PD VS Anxiety**

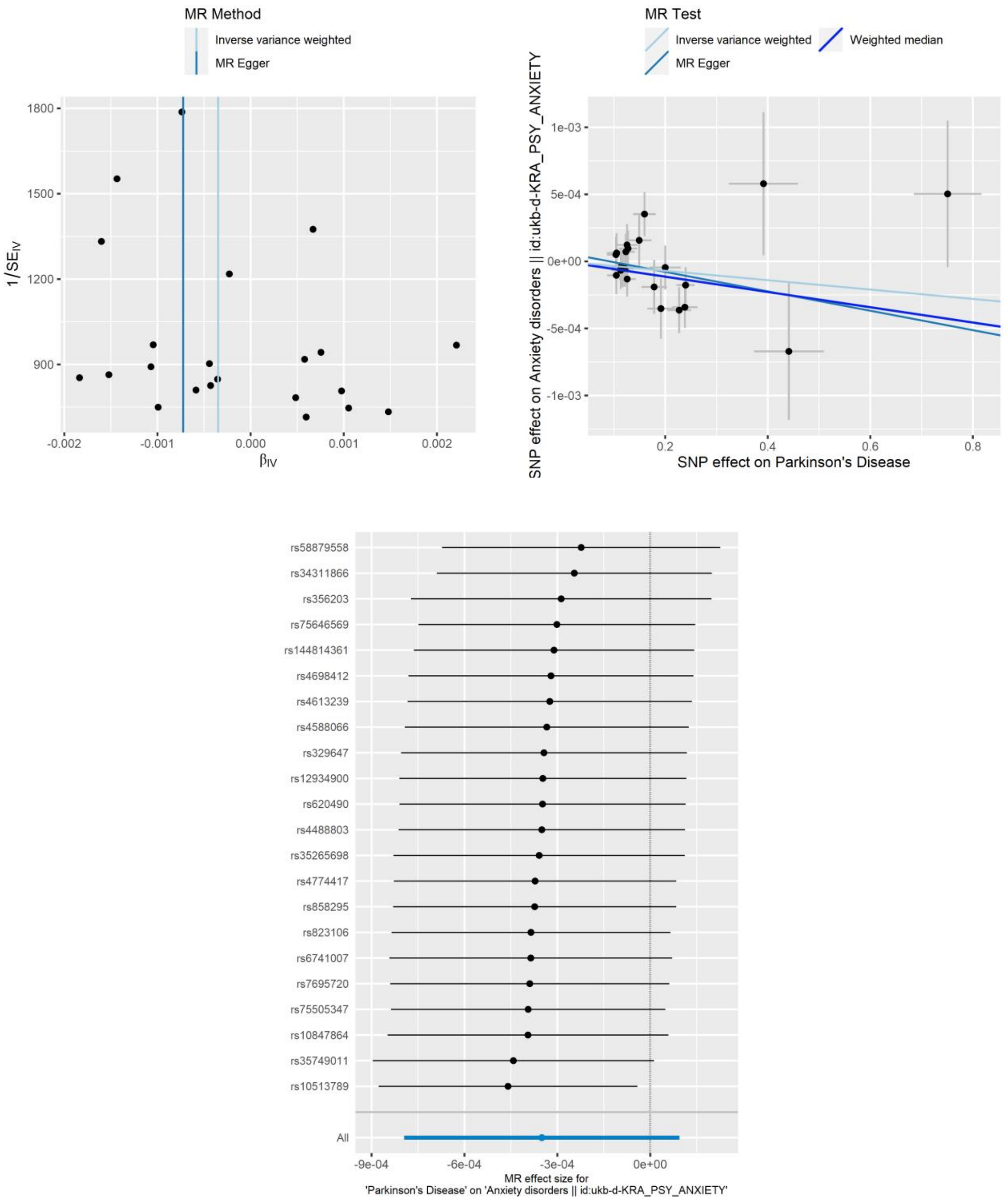

**Supplement Fig.19 PD VS Insomnia**

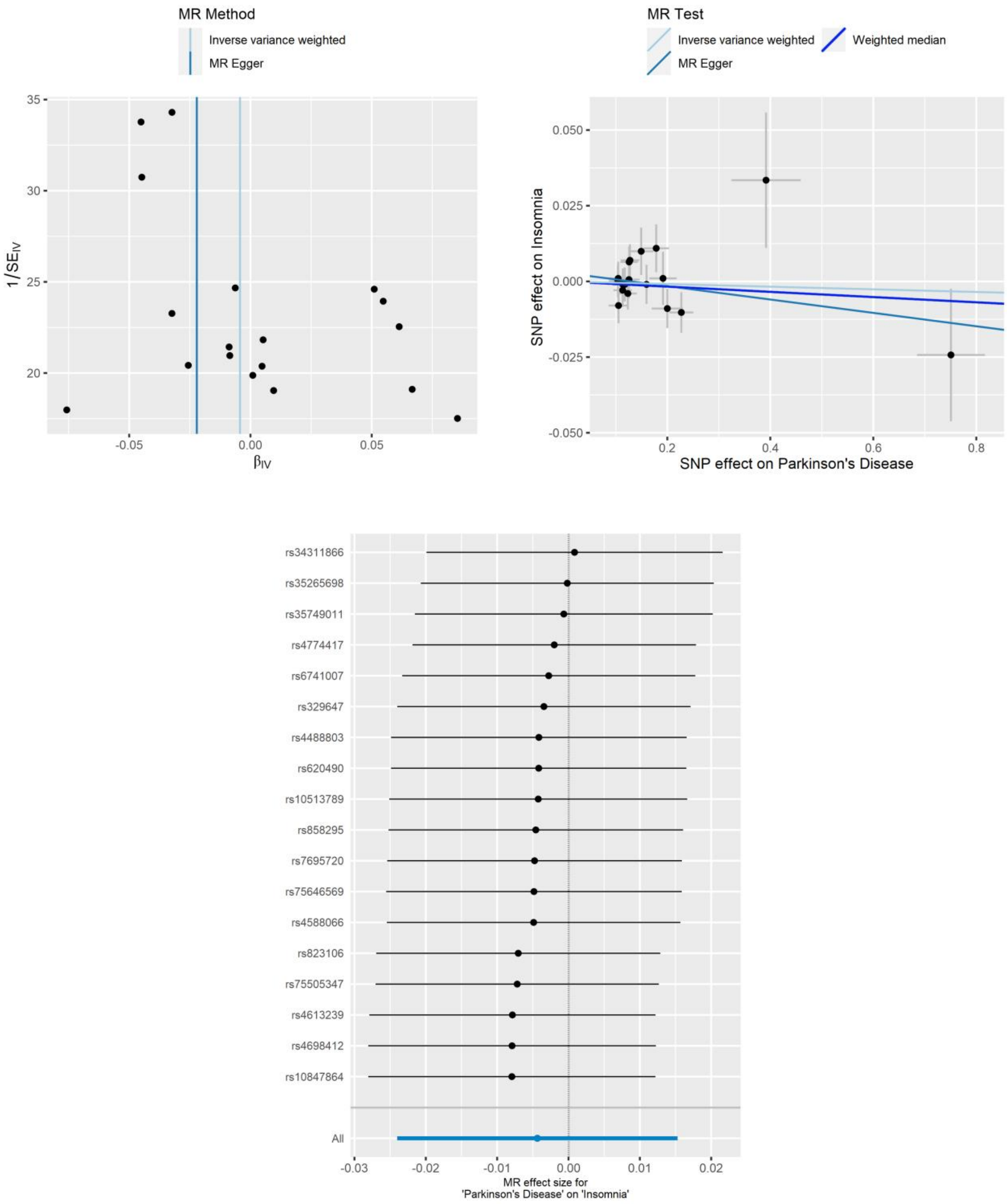

# Supplement Fig. 20 PD VS MDD

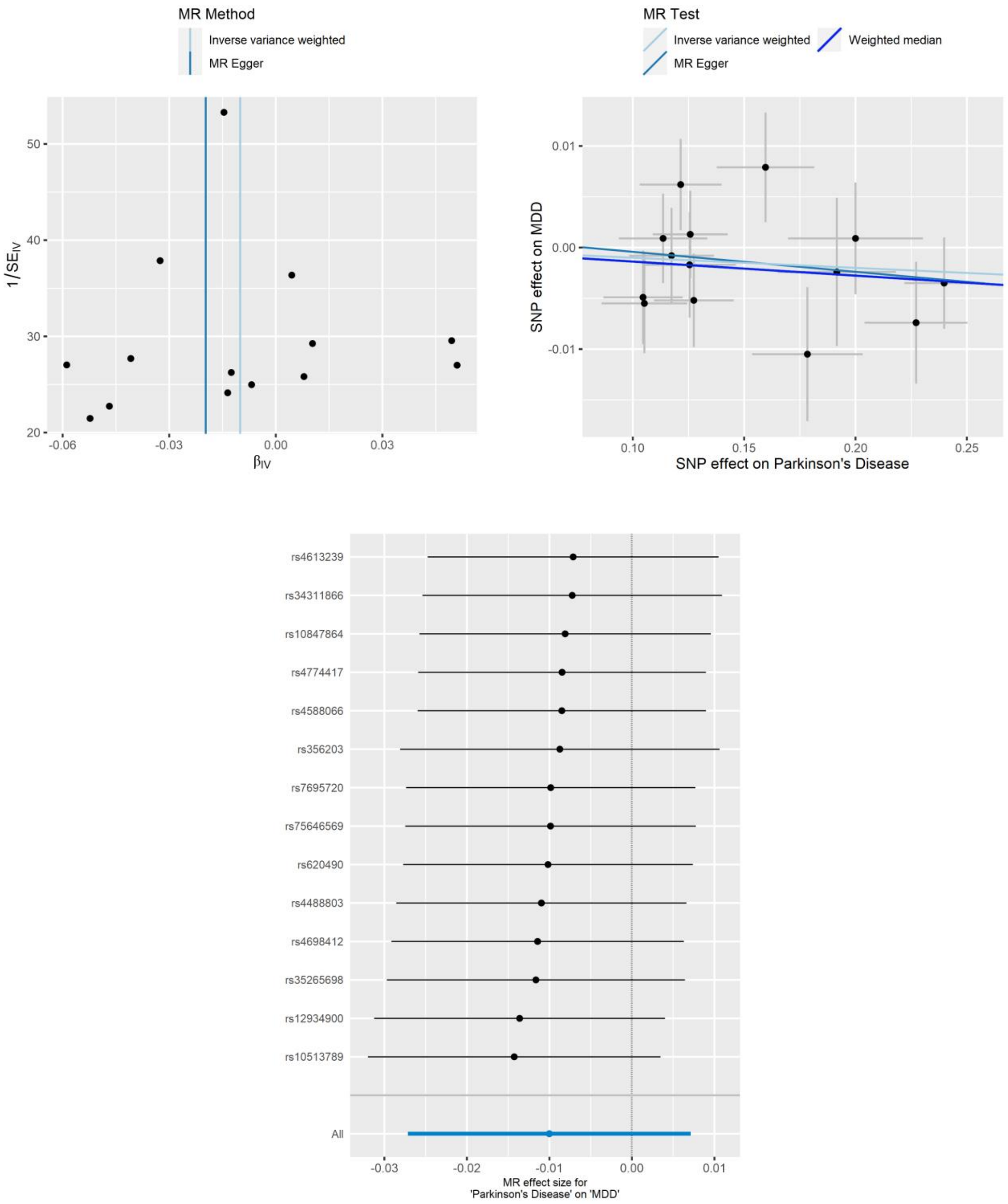

# Supplement Fig.21 PD VS Neuroticism

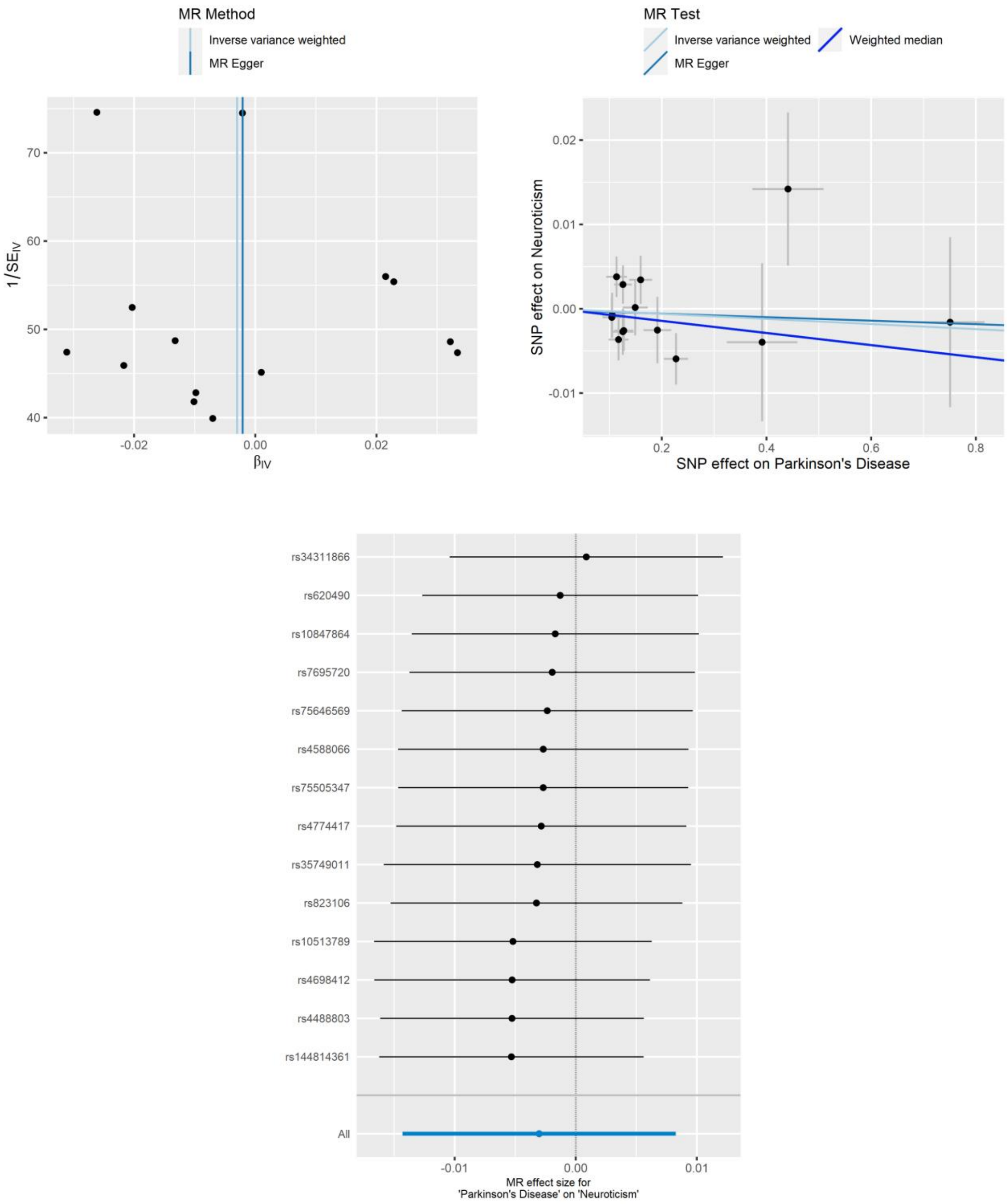

**Supplement Fig.22 PD VS OCD**

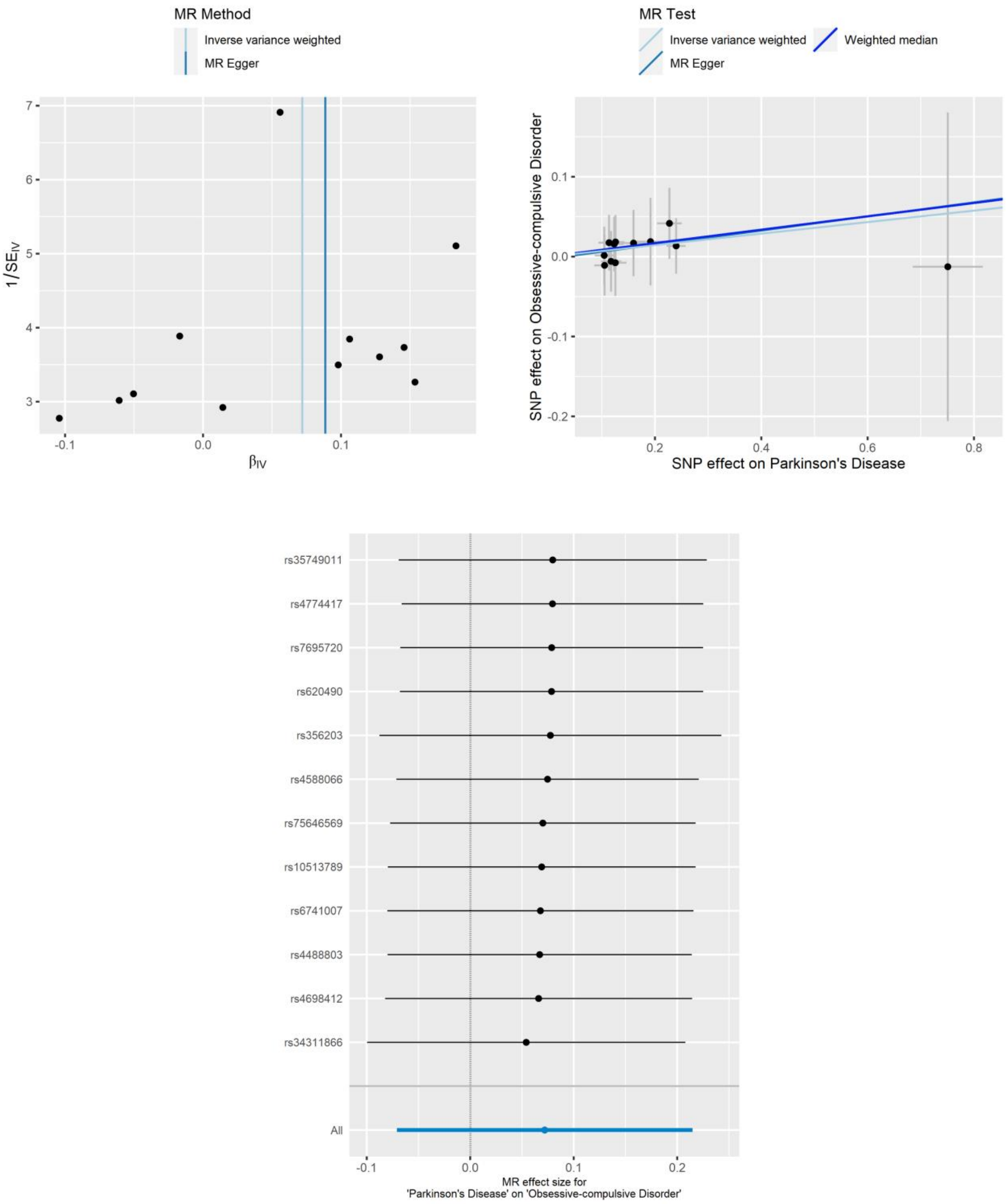

**Supplement Fig.23 PD VS Schizophrenia**

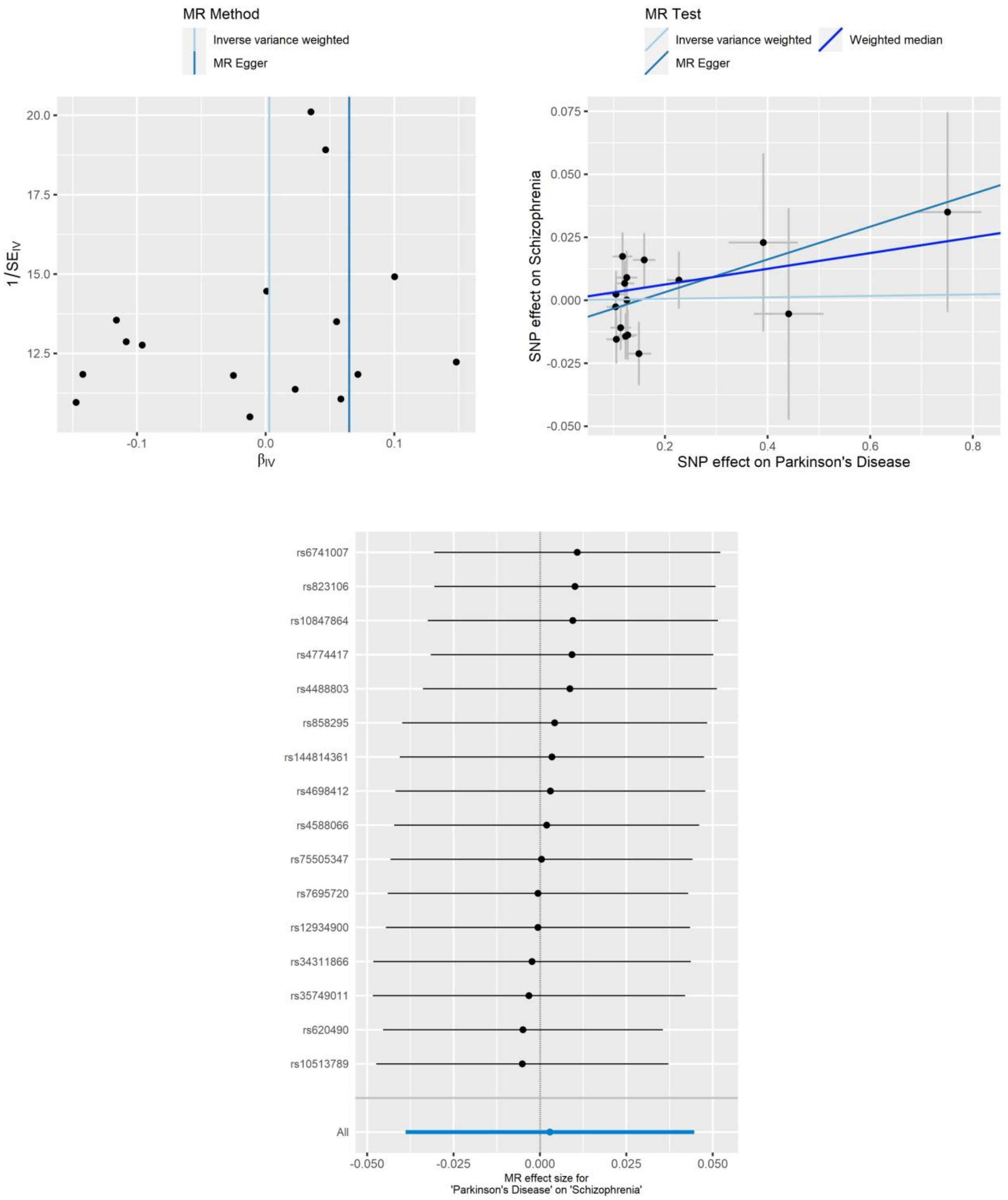

Supplement: Supplementary file 2 [file Data_Sheet_1.PDF]
