# Supplementary material for: Bidirectional Mendelian randomization study of psychiatric disorders and Parkinson’s disease
Source: Front Aging Neurosci. 2023 Mar 14;15:1120615. doi: 10.3389/fnagi.2023.1120615 (PMC10045982; doi:10.3389/fnagi.2023.1120615)
Supplement: Supplementary file 3 [file Data_Sheet_2.PDF]

Part 1. Data presentation

Anorexia Nervosa: The AN GWAS meta-analyzed 33 datasets comprising 16,992 cases and 55,525 controls of European ancestry. The definition of AN was constructed from clinical records and interviews, or online questionnaires based on standardized criteria (Diagnostic and Statistical Manual of Mental Disorders (DSM) III-R, DSM-IV, International Classification of Diseases (ICD) 8, ICD-9 or ICD-10). Eight genome-wide significant loci were identified and 7 of them proved to be independent after conditioning on associated variants discovered in other traits GWAS such as body-mass index (BMI), type 2 diabetes, education years, et al. Full summary statistics was available for downstream analysis. However, no effect allele frequency was provided which is important for the analysis. So when in the forward MR we utilized SNPs eventually reported in the original article.

Anxiety Disorder: Anxiety disorder was defined into two phenotypes in the corresponding GWAS. And we treated the primary phenotype, lifetime anxiety disorder, as exposure. Participants were individuals who took part in the UK Biobank (UKB), a prospective cohort study of over 500 000 people in the United Kingdom. Two definitions were set up and participants who met one of them after discarding those who self-reported a lifetime diagnosis of disorders that are higher heritable than anxiety disorders (schizophrenia, BD, autistic spectrum disorder, attention deficit hyperactivity disorder, or eating disorders) were retained as cases. The first definition was self-reported a lifetime professional diagnosis of one of the core five anxiety disorders and the second was meeting the criteria for a likely lifetime diagnosis of DSM-IV generalized anxiety disorder. Finally, 25, 453 cases (66% of which were female) and 58,113 controls were included. Since summary statistics of this GWAS was not available, we adopted the 5 genome-wide significant loci generated from this GWAS as the primary instrumental variants.

Bipolar Disorder: 57 BD cohorts, totaling 41,917 cases and 371,549 controls of European descent, were included in this GWAS meta-analysis. Cases all met international diagnostic criteria for lifetime BD and controls were screened for the absence of psychiatric disorders via ICD codes. 64 independent loci were identified to be associated with BD at genome-wide significance. GWASs of BD subtypes (bipolar disorder type I and bipolar disorder type II) were conducted separately. (BD I, BD II)44 loci were identified genome-wide significant for bipolar disorder type I (BD I), 31 of which overlapped with those from the main BD GWAS and only one genome-wide significant locus was identified in the GWAS meta-analysis of bipolar disorder type II (BD II) not overlapping with loci from the main BD GWAS. We here take the main BD GWAS as our primary trait for BD.

Insomnia: The largest insomnia GWAS so far with a sample size over 2 million (593,724 cases and 1,771,286 controls) identified 554 risk loci. Subjects included in this study were from UKB and 23andMe. Insomnia was assessed in UKB using a single question that separated cases and controls by dichotomized answers and participants from 23andMe were defined based on multiple questions completed online. Separated GWASs conducted within each cohort discovered 14 and 477 risk loci correspondingly. Taking this gap into account, we utilized the set of independent significant SNPs discovered from the meta-analysis of the 2 cohorts above to gain more power of detecting the true causal estimation.

Major Depressive Disorder: The original MDD GWAS combined three large MDD GWASs before using 807,553 individuals (246,363 cases and 561,190 controls) from 23andMe, UK

Biobank and Psychiatric Genomics Consortium (PGC). Different definitions about MDD were adopted in these cohorts, self-reported diagnosis or treatment for depression for 23andMe, clinically-derived phenotypes MDD for PGC and broad definition of depression for UKB. 102 independent variants were identified and 87 of them were replicated. Full summary-level data was only provided for meta-analysis of PGC and UKB containing 170,756 cases and 329,443 controls.

Neuroticism: We included neuroticism as a primary psychiatric trait for it is the risk factor of many other psychiatric disorders and we wonder if such a trait would cause the risk of PD. The original GWAS was comprised of cohorts from 4 consortiums. Each of them has their own way of measurement of neuroticism. However, available GWAS summary-level data only included participants from UKB that define neuroticism with 12 dichotomous (yes or no) items of the Eysenck Personality Questionnaire Revised Short Form (EPQ-RS). Then the authors calculated a weighted sum-score by adding up individual valid item responses and dividing that sum by the total number of valid responses. After excluding invalid responses (<10 items answered), 390,278 participants were left for GWAS.

Obsessive-compulsive Disorder: All 2688 cases from the OCD GWAS met DSM-IV criteria for OCD. With 7037 genomically matched controls of European ancestry, the GWAS was conducted but no SNPs had reached genome-wide significance. To preliminary explore the causal relationship between OCD and PD, we assumed a suggestive genome-wide significance threshold at $p<1\times10^{-5}$ for the following analysis.

Schizophrenia: The primary GWAS of schizophrenia was a cross-ethnic meta-analysis that combined cohorts of European (EUR), East Asian (ASN) African American (AA) and Latino (LAT) ancestry, totaling 74,776 cases and 101,023 controls. Separate GWASs for male and female individuals were also conducted. 287 distinct genomic loci were detected. From the purpose of the research, we chose the results of combined-sex European individuals as our primary exposure which held 53,386cases and 77,258 controls.

Parkinson's Disease: Two PD GWAS were included in this MR study for replication purpose. The largest PD GWAS so far was regarded as outcome trait in the discovery analysis. Cases from these cohorts were diagnosed through clinic visit and standard UK Brain Bank criteria with a modification to allow the inclusion of cases that had a family history of PD or self-reported diagnosis of PD excluding those with history that could cause parkinsonism. Also, proxy cases defined by family history were included in UKB cohort. Meta analyzing 17 cohorts, the study reported 90 independent genome-wide significant risk signals. FinnGen is a large public-private partnership aiming to collect and analyze genome and health data from 500,000 Finnish biobank participants. The main release of PD GWAS results of FinnGen (R6) was downloaded for replication.
